# A network-based pathway-expanding approach for pathway analysis

**DOI:** 10.1186/s12859-016-1333-x

**Published:** 2016-12-23

**Authors:** Qiaosheng Zhang, Jie Li, Haozhe Xie, Hanqing Xue, Yadong Wang

**Affiliations:** 1 School of Computer Science and Technology, Harbin Institute of Technology, West Da-Zhi Street, Harbin, China; 2 College of Science, Heilongjiang Bayi Agricultural University, Xinfeng Road, Daqing, China

**Keywords:** Pathway analysis, Network-based, Significant pathway, Protein-protein interaction

## Abstract

**Background:**

Pathway analysis combining multiple types of high-throughput data, such as genomics and proteomics, has become the first choice to gain insights into the pathogenesis of complex diseases. Currently, several pathway analysis methods have been developed to study complex diseases. However, these methods did not take into account the interaction between internal and external genes of the pathway and between pathways. Hence, these approaches still face some challenges. Here, we propose a network-based pathway-expanding approach that takes the topological structures of biological networks into account.

**Results:**

First, two weighted gene-gene interaction networks (tumor and normal) are constructed integrating protein-protein interaction(PPI) information, gene expression data and pathway databases. Then, they are used to identify significant pathways through testing the difference of topological structures of expanded pathways in the two weighted networks. The proposed method is employed to analyze two breast cancer data. As a result, the top 15 pathways identified using the proposed method are supported by biological knowledge from the published literatures and other methods. In addition, the proposed method is also compared with other methods, such as GSEA and SPIA, and estimated using the classification performance of the top 15 expanded pathways.

**Conclusions:**

A novel network-based pathway-expanding approach is proposed to avoid the limitations of existing pathway analysis approaches. Experimental results indicate that the proposed method can accurately and reliably identify significant pathways which are related to the corresponding disease.

**Electronic supplementary material:**

The online version of this article (doi:10.1186/s12859-016-1333-x) contains supplementary material, which is available to authorized users.

## Background

Complex diseases are likely to be associated with the effects of multiple genes, proteins and biological pathways [1]. Pathway analysis methods that combine multiple types of high-throughput data, such as genomics and proteomics, have become the first choice to gain insights into the pathogenesis of complex diseases. A biological pathway that reduces data involving thousands of altered genes and proteins into a smaller and more interpretable set of altered processes and combines multiple types of high-throughput data plays an important role in understanding the mechanisms of complex diseases, improving clinical treatment, and discovering drug targets and biomarkers [2].

The most commonly employed traditional pathway analysis methods use classical pathway databases (i.e., KEGG [3], MSigDB [4], Reactome [5], BioCyc [6], MetaCyc [7], RegulonDB [8], PantherDB [9] and Gene Ontology [10]) to analyse gene expression profile data. These analyses use statistical methods to identify significant pathways in a particular biological process, such as GSEA [11], PAGE [12], GAGE [13] and MeanAbs [14]. A limitation of this class of algorithms is their ignorance of interactions between genes and proteins because neither network topology nor dynamics is taken into account [15]. These limitations are addressed by network-based pathway analyses. Accordingly, several pathway analysis models that reflect the laws of life activities and employ network topology information have been proposed [16], such as SPIA [17], PARADIGM [18], PathOlogist [19], Active Modules [20], AMBIENT [21], GIGA [22] and GANPA [23]. Although the above methods utilize network topology information, they only consider the topological structure of the pathway itself and do not take into account the information of pathway external genes in biological networks; thus, they do not fully mine pathway information. For example, only the pathway internal topology is utilized by the SPIA method, whereas the PathOlogist model only computes the probability of an interaction of pathway internal genes being active when it is consistent with the known regulatory logic of the pathway. Hence, how to take the interactions between internal and external genes of the pathway and between pathways into account in the pathway analysis method is the main problem addressed in this paper.

**Fig. 1:**
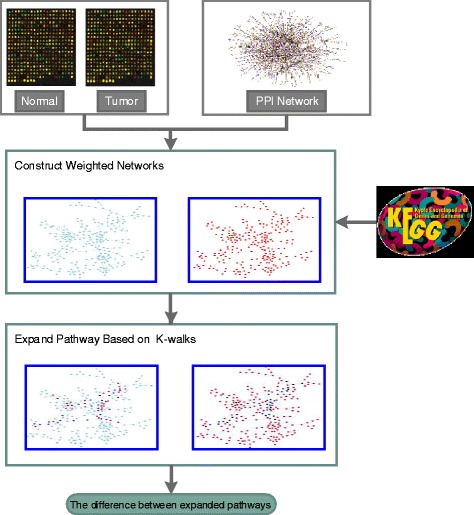
Workflow of the proposed method

To that end, we proposed a novel network-based pathway analysis method. First, we integrated protein-protein interaction (PPI) information, gene expression profile data and pathway databases into the pathway analysis and constructed two whole-genome level gene-gene interaction networks. Then, we expanded pathways based on the k-walks algorithm [24, 25] to two small networks in two weighted networks (tumor and normal). Finally, we scored the pathways corresponding to the gene expression profile data based on the correlations of these two small networks to identify significant pathways (see Fig. 1).

## Methods

### Construction of a weighted gene-gene interaction network

PPI network provides a valuable framework to elucidate the functional organization of the proteome. However, existing PPI networks cannot accurately describe the interactions between proteins in specific conditions and have different degrees of false positive and false negative results because most large-scale PPI networks are obtained in different experimental conditions, predicted/extracted using different algorithms [26, 27]. Additionally, the interaction or the intensity between proteins varies in different cells or tissues.

The gene co-expression network (GCN) is an undirected graph where each node corresponds to a gene and a pair of nodes is connected with an edge if there is a significant co-expression relationship between them [28]. Using gene expression profiles obtained from a number of genes for several samples or experimental conditions, a gene co-expression network can be constructed by looking for pairs of genes that show a similar expression pattern across samples. In this study, the weight of each pair of genes is calculated by the Pearson’s correlation coefficient. Pearson’s correlation coefficient was selected as the co-expression measurement because it was the most popular co-expression measurement used in the construction of gene co-expression networks. The absolute values correspond to an interaction mechanism where the intensity of one gene is related to its co-expressed gene. However, a gene co-expression network does not guarantee the existence of a real interaction between the corresponding proteins; instead, it only suggests that there may be an interaction between the proteins.

**Fig. 2:**
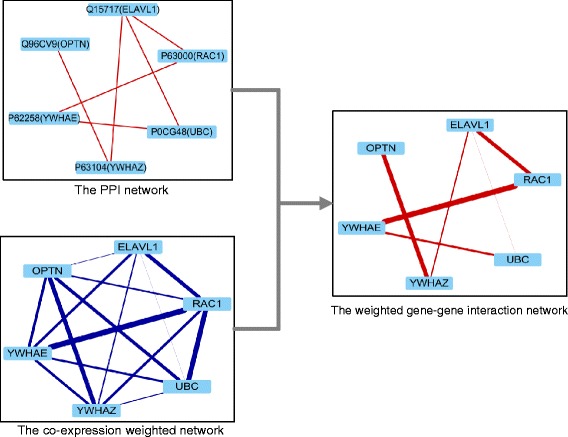
Construction of the weighted gene-gene interaction network (the edge width reflects weight size in the weighted gene-gene interaction network). The PPI network comes from I2D, the co-expression weighted network is from gene expression profiling, and the weight of each pair of genes is calculated by Pearson’s correlation coefficient. Finally, the PPI network and the co-expression weighted network are merged into the weighted gene-gene interaction network. We obtain two weighted gene-gene interaction networks under two phenotype datasets (tumor and normal)

To accurately describe the change in gene interactions for several samples or experimental conditions, here we constructed two weighted gene-gene interaction networks (tumor and normal) with PPI and GCN (see Fig. 2).

### Pathway-based extension of the sub-network

The gene-gene interaction of pathway is different in different tissues or samples. These differences may be caused by changes in the interactions between internal genes of the pathway or between pathway and neighbor genes. To assess the significance of the pathway in different phenotypic data, we expanded the pathway based on the k-walk algorithm [25] by considering all of the above factors in two weighted gene-gene interaction networks separately. The pathway-based extension of the sub-network was constructed as follows:

Let *G*=(*V,E*) comprise a set *V* of genes and a set *E* of edges denote the weighted gene-gene interaction network with *E*⊆*V*×*V*. Let *n*=∣*V*∣reflect the number of genes. Symmetric matrix *A* represents the weighted *n*×*n* adjacency matrix of *G*, where *a*_*ij*_denotes the weight of the edge connecting gene *i* to gene *j*. Let *d*_*i*_(*i*∈1⋯*n*) represent the weighted degree of each gene node *i* where $d_{i}=\sum ^{n}_{j=1}a_{ij}$. Then, *a*_*ij*_ is calculated by Pearson’s correlation coefficient as:

1 $$ a_{ij}=\left\{ \begin{array}{ll} |cor(x_{i},x_{j})|^{\beta} &{x_{i},x_{j} \ is \ expression} \\ & { data \ of \ gene \ V_{i},V_{j}}\\ 0 & otherwise \end{array}\right.  $$

where *β*=1.

Given a gene set *S* (|*S*|≥2) of a pathway belonging to a subset of *G*, we formally define an edge relevance function *ER*:$E\rightarrow \mathbb {R^{+}}$ that maps any edge to its relevance. The extended process of a gene set of a pathway simulates random walks on a graph by the Markov Chain model. The possibility of transiting from gene *i* to gene *j* is calculated as:

2 $$ P_{ij}=\frac{a_{ij}}{d_{i}}  $$

Here, a gene set *S* is a set of absorbing states of the Markov chain. If the random walk starts from gene *x*, the modified transition will be:

3 $$ {^{x}}P_{ij}=\left\{ \begin{array}{ll} 1 &i \in S \backslash \{x\} \ and \ i=j\\ 0 &i \in S \backslash \{x\} \ and \ i\neq j\\ P_{ij} & otherwise \end{array}\right.  $$

Then, the transition matrix is described as follow:

4 $$ {^{x}}P= \left(\begin{array}{cc} {^{x}}Q & {^{x}}R \\ 0 & I \\ \end{array} \right)  $$

where ^*x*^ *Q* is a matrix that denotes transient states, ^*x*^ *R* is a matrix that denotes the transition probability from transient states to absorbing states, and *I* is the identity matrix. After *k* steps, the transition matrix becomes (^*x*^ *Q*)^*k*^.

Given that the walk started in state *x*, the joint probability of visiting the edge *E*(*i,j*) between step *k* and *k*+1 is calculated as follow:

5 $${} \begin{aligned} &P\left[X_{k}=i,X_{k+1}=j,L|X_{0}=x\right]= \\ &\left\{\begin{array}{lll} f(i,j,k,x,r,L) & j \ is \ a \ transient \ state \\ \left[({^{x}}Q){^{L-1}}\right]{_{xi}}[({^{x}}R) ]{_{ij}} & j \ is \ an \ absorbing \ state \end{array}\right. \end{aligned}  $$

where *L* is a total walk length, $f(\,i,\,j,\,k,\,x,\,r,\,L)\,= \!\!\!\!\!\mathop {\sum }\limits _{r\in S \backslash \{x\}}[({^{x}}Q){^{k}} ]{_{xi}}[({^{x}}Q)]{_{ij}} [({^{x}}Q){^{L-k-2}}({^{x}}R) ]{_{jr}}$, [(^*x*^ *Q*)^*k*^]_*xi*_ is the probability of transiting from *x* to *i* in *k* steps.

The probability of a walk of length *L* starting in *x* is calculated as follow:

6 $$ P[L|X{_{0}}=x]=\mathop{\sum}\limits_{r\in S \backslash \{x\}}[({^{x}}Q){^{L-1}}({^{x}}R) ]{_{xr}}  $$

The *e*(*x,i,j*) is defined as the number of times a random walk starts in *x* using the transition from *i* to *j*. Given that the walk length is *L*, the conditional expectations of *e*(*x,i,j*) is given by:

7 $$ E[e(x,i,j)|L]=\sum_{k=0}^{L-1}\frac{P[X_{k}=i,X_{k+1}=j,L|X_{0}=x]}{P[L|X_{0}=x]}  $$

Let *L*_*max*_ denote a maximal walk length. Then:

8 $$ E[e(x,j,i)|L\leq L_{max}]=\sum_{L=1}^{L_{max}}E[e(x,i,j)|L]  $$

**Fig. 3:**
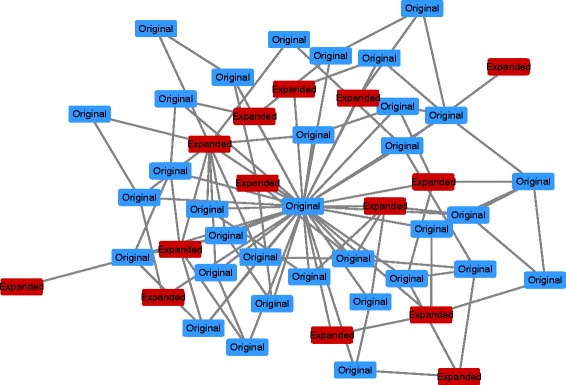
An example of a pathway-based extension. *Blue nodes* denote a gene set of a pathway and *red nodes* denote the expanded genes that are most associated with the corresponding pathway

Finally, the edge relevance *ER* is given by:

$${} ER(i,j)= $$

9 $$ \begin{aligned} &\mathop{\sum}\limits_{r\in S}l_{x}|E\left[e(x,i,j)|L\leq L_{max}\right]- E\left[e(x,j,i)|L\leq L_{max}\right] | \ \forall(i,j)\in E \end{aligned}  $$

where the vector *l*_*x*_ represents an initial probability distribution. Here, the maximal relevance score that can lead to a connected subgraph is chose as the threshold *θ*. Finally, a subnetwork is obtained by keeping only edges with relevance scores *ER*(*i,j*) above a threshold value *θ* (see Fig. 3). In the paper, we set *L*_*max*_ to 50 by default.

### Identification of significant pathways

For a given pathway *i*, the pathway was expanded in two weighted gene-gene interaction networks (Tumor_Net and Normal_Net)for two phenotypic datasets separately. The union genes of two expanded pathway play a role in performing a similar function in normal or tumor tissues. Moreover, genes in expanded pathway from tumor and normal tissues are almost different. The union genes construct sub-networks in two weighted networks. These sub-networks weights’ differences can describe the change of pathways between different phenotypes. Accordingly, we calculate the difference between the two pathway-based sub-networks reflects the change of the given pathway for the two phenotypic datasets through the union of the two sub-networks.

Let Union_Pathway [*i*] denote the union of two sub-networks (T_Ex_Pathway [*i*] and N_Ex_Pathway [*i*]) that are the expansion of pathway *i* in two weighted gene-gene interaction networks (Tumor_Net and Normal_Net). Then, we mapped Union_Pathway [*i*] into the two weighted gene-gene interaction networks (T_subnet [*i*] and N_subnet [*i*]) and obtained two edge weight vectors T_w [*i*] and N_w [*i*]. Pearson’s correlation coefficient was calculated as:

$${} Corr_{i}(T\_w[i],N\_w[i])= $$

10 $$ \begin{aligned} \frac{\sum_{k=1}^{n}(T\_w[i]_{k}-\overline{T\_w[i]})(N\_w[i]_{k}-\overline{N\_w[i]})}{\sqrt{\sum_{k=1}^{n}(T\_w[i]_{k}- \overline{T\_w[i]})^{2}\sum_{k=1}^{n}(N\_w[i]_{k}-\overline{{N\_w[i]}})^{2}}} \end{aligned}  $$

where *n* is the dimension of the vector and $ \overline {T\_w[i]}= \frac {1}{n}\sum _{k=1}^{n}T\_w[i]{_{k}}$, $ \overline {N\_w[i]}= \frac {1}{n}\sum _{k=1}^{n}N\_w[i]{_{k}}$. Finally, we calculated Score [*i*], which depicts the difference in pathway *i* for two phenotypic datasets as follow:

11 $$ Score[i]=1-|Corr_{i}(T\_w[i],N\_w[i])|  $$

Here, Score [*i*] is a measure depicting the relevance degree between pathway *i* and the corresponding disease (for the pseudo-code see Algorithm 1).

## Results and discussion

### Data

The breast invasive carcinoma (BRCA) dataset was downloaded from the TCGA (The Cancer Genome Atlas) website (http://cancergenome.nih.gov/). The BRCA dataset consists of 590 samples obtained from comparing 529 breast cancer samples with 61 normal samples using the Agilent platform. The second dataset was available via the Gene Expression Omnibus (ID= GSE25066). This dataset compared 99 pathologic complete response (PCR) samples and 389 residual disease (RD) samples [29] (http://www.ncbi.nlm.nih.gov/geo/query/acc.cgi?acc=GSE25066). PPI network (version 2.9) was obtained from the Interologous Interaction Database (I2D) website [26] (http://ophid.utoronto.ca/ophidv2.204/downloads.jsp). PPI was mapped into the gene-gene interaction (GGI) data through the UniProt website (www.uniprot.org/). Finally, 234,524 unique gene pairs were selected for BRCA, and 204,772 unique gene pairs were selected for GSE25066 by data pretreatment. The KEGG pathways were downloaded from the ConsensusPathDB website (http://consensuspathdb.org/). We selected 280 pathways related to humans by screening; only genes of pathways belonging to the BRCA gene set were used in the downstream analysis. The breast cancer gene set was downloaded from website(http://mlg.hit.edu.cn/SIDD/).

To identify the significance of the given pathway, first, we dealt with the PPI data. The PPI network was mapped into the gene-gene interaction (GGI) network in which the weight of each pair of genes was calculated using high-throughput gene expression profiling data. Finally, we obtained two weighted gene-gene interaction networks for the two phenotypic datasets. The weighted gene-gene interaction network has 15,129 vertices and 234,524 edges for BRCA.

**Fig. 4:**
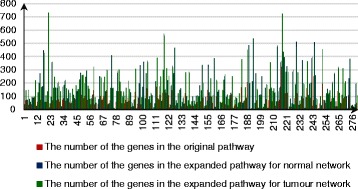
The number of the genes in the original pathway and the expanded pathway. Through the diagram, we found that every pathway was validly expanded except pathway hsa00472 because it only contained one gene from the original pathway

Based on the above algorithm, we expanded a gene set of the given pathway based on the k-walks algorithm into two sub-networks in two weighted networks (tumor and normal). Then, we compared the number of the genes in the original pathway and the expanded pathway (see Fig. 4). Finally, the union of the two sub-networks served as the ultimate expansion of the given pathway.

Next, we ran the proposed approach using the BRCA dataset.

To provide a more comprehensive understanding of the proposed method, we discuss the method from the following aspects separately.



### The pathway score

Based on the background mentioned above, each pathway score depicts the degree of relevance between the given pathway and the corresponding disease. All scores were calculated using Algorithm 1 (Additional file 1: Table S1). The top 15 pathways were tabulated based on their scores (see Table 1).

**Table 1:** Top 15 pathways identified from BRCA

Rank	Entry	Name	Score	SPIA	GSEA	Proof
1	hsa00750	Vitamin B6 metabolism	0.997735	No	No	[30–33]
2	hsa00072	Synthesis and degradation of ketone bodies	0.940425	No	Yes	[34–37]
3	hsa04122	Sulphur relay system	0.855753	No	No	[38, 39]
4	hsa00400	Phenylalanine,tyrosine and tryptophan biosynthesis	0.850563	No	No	[40]
5	hsa00533	Glycosaminoglycan biosynthesis	0.836469	No	No	[41–43]
6	hsa04964	Proximal tubule bicarbonate reclamation	0.803311	No	Yes	[46]
7	hsa01040	Biosynthesis of unsaturated fatty acids	0.799334	No	No	[47]
8	hsa00630	Glyoxylate and dicarboxylate metabolism	0.785954	No	Yes	[48]
9	hsa05217	Basal cell carcinoma	0.779876	No	No	[49, 50]
10	hsa00910	Nitrogen metabolism	0.77962	No	Yes	[51, 52]
11	hsa05218	Melanoma	0.758975	Yes	Yes	[53]
12	hsa04972	Pancreatic secretion	0.754263	No	Yes	[54, 55]
13	hsa00670	One carbon pool by folate	0.7452	No	No	[56]
14	hsa00900	Terpenoid backbone biosynthesis	0.736641	No	No	[57, 58]
15	hsa00920	Sulphur metabolism	0.733627	No	No	[59]

Note: Yes if the pathway was also ranked in the SPIA or GSEA top 15; No if otherwise

**Fig. 5:**
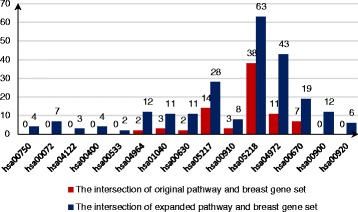
Comparison between the gene number of intersections of the breast gene set and pathway gene sets before and after expansion

Top pathways identified by the proposed method should be significantly associated with the breast cancer risk. To test the idea, we compared the intersections of the breast cancer gene set and pathway gene sets before and after expansion (see Fig. 5). The breast cancer gene set comes from SIDD which integrates 22 disease gene knowledge sources. We found that more genes associated with breast cancer were expanded to the original pathway gene set through pathway expansion. This result demonstrates that the proposed method can expand genes associated with the corresponding disease.

### Analysis of the top 15 BRCA pathways

In order to prove that the pathways identified by the proposed method are associated with the breast cancer risk, we need to look for the supports of biological knowledge and other methods. Table 1 shows that top 15 pathways identified from BRCA by the proposed method are significantly associated with the breast cancer risk through reference. Here we give the supports of biological knowledge and other methods for the top 15 BRCA pathways.

The number 1 ranked significant pathway identified by our method was vitamin B6 metabolism (hsa00750). Growing evidence suggests that the lack of several trace elements, such as vitamin B6 and folate, can induce DNA damage (e.g., single or double-stranded breaks or fusion), eventually leading to tumors, cancers and a variety of degenerative diseases [30]. There is a significant negative correlation between the plasma B6 level and different types of cancer. Vitamin B6 can reduce the homocysteine and pyridoxal phosphate levels, which have potential biological effects on tumors. Vitamin B6 deficiency leads to lower serine hydroxymethyltransferase activity, lower generation of 5,10-methylenetetrahydrofolate and the generation of a dUMP instead of a dTMP mismatch to DNA, which is more likely to lead to a chromosome chain break and /or impair DNA excision repair. The reduced generation of 5,10-methylenetetrahydrofolate may lead to DNA hypomethylation. Abnormal methylation of DNA has been found in different tumor types [31, 32]. Vitamin B6 deficiency can increase the sensitivity of the steroid hormone,which may lead to breast cancer or colon cancer [33]. These findings demonstrate that the proper intake of vitamin B6 can reduce the risk of breast cancer; therefore, this pathway is significantly associated with the breast cancer risk.

The number 2 ranked significant pathway identified by the proposed method was Synthesis and degradation of ketone bodies (hsa00072). Ketone bodies (i.e., 3-hydroxy-butyrate and/or butanediol) are sufficient to drive mitochondrial biogenesis in human breast cancer cells [34, 35]. Carcinoma-associated fibroblasts (CAF) produce “mitochondrial fuels”, including lactic acid, ketones, fatty acids, and glutamine, that provide a "eutrophication" microenvironment for tumor cells and promote tumor cell proliferation when metabolized; these fuels are the major cellular components of the breast cancer stroma [36]. It was reported that CAF reduced the apoptosis of human breast cancer MCF7 cells induced by tamoxifen and fulvestrant by 4.4-fold and 2.5-fold, respectively [37]. Lactic acid and ketones are sufficient to induce tamoxifen resistance in breast cancer MCF7 cells. Metformin and arsenic trioxide can overcome CAF-induced drug resistance in MCF7 cells. These findings indicate that this pathway is also significantly associated with the breast cancer risk.

The proposed method ranked the Sulphur relay system (hsa04122) in 3rd place. Sulphur enables the transport of oxygen across cell membranes. Oxygen is necessary for healthy cellular regeneration in mammals. Therefore, sulphur deficiencies may promote sickness and disease. Sulphur is commonly used as an herbal medicine to treat inflammation and cancer and organic sulphur has been studied in several types of cancers and found to have remarkable anti-cancer benefits. Methylsulfonylmethane (MSM) is an organic sulphur-containing natural compound without any toxicity. It was found that MSM substantially decreased the viability of human breast cancer cells in a dose-dependent manner and recommended the use of MSM as a trial drug for the treatment of all types of breast cancers [38]. Leimkühler et al. pointed out that sulphur not only prevented but also helped reverse cancer [39]. Hence, the sulphur relay system is significantly associated with the breast cancer risk to some extent.

Phenylalanine, tyrosine and tryptophan biosynthesis (hsa00400) was ranked 4th in the list of proposed methods. ENO1 in phenylalanine tyrosine and tryptophan biosynthesis was significantly overexpressed in HER-2/neu positive breast tumors [40]. This finding indicates that this pathway is associated with breast cancer to some extent; however, the clear relationship between this pathway and breast cancer re-quires further verification.

The 5th ranked pathway was Glycosaminoglycan biosynthesis (hsa00533). Abnormal glycosaminoglycan (GAG) concentrations have been reported for various types of tumors, suggesting that they may play a role in neoplasia. Recently, cell biology studies revealed that glycosaminoglycans were among the key macromolecules that affected cell properties and functions by acting directly on cell receptors or via interactions with growth factors. The interactions of GAGs with growth factors, cytokines and growth factor receptors have been implicated in cancer growth and progression. GAGs are involved in signalling cascades that regulate the angiogenesis, invasion and metastasis of malignant cells. Investigations of the fine structures and specific biological roles of GAGs has led to novel therapeutic approaches [41–43]. The above references denote that glycosaminoglycan biosynthesis and breast cancer have a certain degree of correlation.

The top 6–15 pathways are also associated with human breast cancer (see Table 1). Based on the Table 1, one can argue that the proposed method is very efficient in identifying significant pathways of the corresponding complex disease.

### Classification performance using the original genes and expanded genes of the pathway

**Fig. 6:**
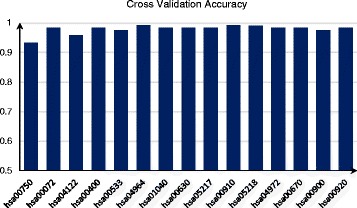
Cross validation accuracy using 10-fold cross validation

To estimate the classification performance of the top 15 expanded pathways, we firstly prepared our data set consisted of 60 normal and 60 tumor samples randomly derived from the BRCA dataset. The original genes and expanded genes of the pathway were selected classification features and SVM is employed to classify the selected samples. Next, a 10-fold cross validation was used to train and test SVM. The above experiment was repeated 100 times and the average accuracy of SVM is shown in Fig. 6.

In results, the lowest accuracy was 0.9333 and the highest accuracy was 0.9917. The experimental results suggested that the union of the original genes and expanded genes of the pathway had a good classification ability and that the top 15 pathways were significantly different between the two phenotypic data sets.

### Analysis using alternative methods

To assess the validity of the proposed approach, we analysed the same data using GSEA and SPIA. The GSEA approach searches for gene sets that are enriched at the top or bottom of the ranked list of all genes. This method is a typical representative of the gene set enrichment analysis methods. The SPIA method scores a gene product as highly impactful if it points to other impactful gene products in the network diagram. This method is a representative of the network-based pathway analysis approaches. Therefore, we compared our method with GSEA and SPIA. It was interesting to examine pathways ranked at the top by our method but not by GSEA and SPIA, which reflected the validity of our method.

In GSEA, the analysis performed 1000 permutations with an FDR cutoff of 25%. Then, 115 pathways were identified (Additional file 2: Table S2) of which 6 were found among the top 15 pathways identified using the proposed method (see Table 1).

In SPIA, a significant threshold of 5% was used on the FDR corrected P-values to infer pathway significance. Then, 3 pathways were identified (Additional file 3: Table S3) of which one was identified by the proposed method (see Table 1). The SPIA did not identify any of the top 5 pathways identified using the proposed method.

### Validation of the alternative data set

To test the effectiveness of the proposed method, we ran the proposed approach on GSE25066. The data were obtained from response and survival following Taxane-Anthracycline chemotherapy for newly diagnosed invasive breast cancer. Anthracyclines and taxanes are the two most active classes of cytotoxic agents for early and advanced stage breast cancer and thus are commonly used as a component of either adjuvant or neoadjuvant therapy and/or in patients with metastatic breast cancer (MBC) [44]. Finally, we also obtained two weighted gene-gene interaction networks for the two phenotypic datasets. The weighted gene-gene interaction network has 10,856 vertices and 204,772 edges for GSE25066. Our intention was to identify significant pathways for breast cancer patients before and after Taxane-Anthracycline use and to evaluate the pharmacological mechanism of Taxane-Anthracycline. Among the top 15 pathways identified using the proposed method, which were significant pathways for Taxane-Anthracycline except for collecting duct acid secretion pathway (hsa04966), the GSEA and SPIA did not identify any. The relationship between collecting duct acid secretion pathway and Taxane-Anthracycline and/or breast cancer requires further verification. The results (Additional file 4: Table S4) showed that our approach discovered significant pathways for Taxane-Anthracycline. The top 15 pathways are shown in Table 2.

**Table 2:** Top 15 pathways identified from GSE25066

Rank	Entry	Name	Score	SPIA	GSEA	Proof
1	hsa05033	Nicotine addiction	0.430656	No	No	[60]
2	hsa05217	Basal cell carcinoma	0.402534	No	No	[49, 50]
3	hsa04740	Olfactory transduction	0.398952	No	No	[61, 62]
4	hsa04742	Taste transduction	0.393069	No	No	[63, 64]
5	hsa04340	Hedgehog signaling pathway	0.376035	No	No	[65, 66]
6	hsa04727	GABAergic synapse	0.362031	No	No	[67]
7	hsa04713	Circadian entrainment	0.356687	No	No	[68, 69]
8	hsa00053	Ascorbate and aldarate metabolism	0.35363	No	No	[70]
9	hsa04723	Retrograde endocannabinoid signaling	0.343314	No	No	[71–73]
10	hsa04978	Mineral absorption	0.342461	No	No	[74]
11	hsa04961	Endocrine and other factor-regulated calcium reabsorption	0.341614	No	No	[75–77]
12	hsa00140	Steroid hormone biosynthesis	0.337664	No	No	[78, 79]
13	hsa04966	Collecting duct acid secretion	0.336179	No	No	Not Found
14	hsa04330	Notch signaling pathway	0.333964	No	No	[80, 81]
15	hsa04614	Renin-angiotensin system	0.332994	No	No	[82, 83]

Note: Yes if the pathway was also ranked in SPIA or GSEA top 15; No if otherwise

The significantly impacted pathways identified by the proposed method in the corresponding conditions were mostly consistent with the known biological processes. Accordingly, the novel proposed method is of methodological and biological significance for future research.

### Conclusions

Pathway analysis not only reduces data involving thousands of altered genes and proteins to a smaller and more interpretable set of altered processes but can also combine multiple types of high-throughput data. The analysis results play an important role in elucidating the mechanisms of complex diseases, improving clinical treatment, and discovering drug targets and biomarkers. Therefore, pathway-based analysis of complex diseases has become a research hotspot. To date, these methods have entered the third stage [45]: 1) Pathway-based gene set enrichment analysis; 2) Pathway-based functional class clustering and scoring approaches; and 3) Network-based pathway approaches.

Unlike existing pathway analysis approaches that do not take into account the interaction between internal and external genes of the pathway and between pathways, we propose a novel approach that addresses the above-mentioned limitations by expanding a pathway based on the k-walk algorithm to two small networks in two weighted networks (tumor and normal). Finally, our approach effectively identified significant pathways that corresponded to a complex disease through a series of verification steps. It is undeniable that the pathways identified by GSEA and SPIA but not by our method are mostly significantly associated with the breast cancer risk. Based on the above analysis, our method combined with GSEA may produce better results. Hence, we will combine our method with GSEA in future studies. This study provides a new research direction for the pathway-based analysis of complex diseases. We will employ more datasets to assess the validity of our approach in future research.

## Additional files

Additional file 1 Table S1. The scores of pathways from BRCA. (PDF 234 kb)

Additional file 2 Table S2. The results of GSEA from BRCA. (PDF 321 kb)

Additional file 3 Table S3. The results of SPIA from BRCA (Top 20). (PDF 110 kb)

Additional file 4 Table S4. The scores of pathways from GSE25066. (PDF 272 kb)
