# Supplementary material for: A network-based pathway-expanding approach for pathway analysis
Source: BMC Bioinformatics. 2016 Dec 23;17(Suppl 17):536. doi: 10.1186/s12859-016-1333-x (PMC5259956; doi:10.1186/s12859-016-1333-x)
Supplement: Additional file 1 — Table S1. The scores of pathways from BRCA. (PDF 234 kb) [file 12859_2016_1333_MOESM1_ESM.pdf]

Table S1. The scores of pathways from BRCA

Rank	Number	Entry	Name	Score
1	176	path:hsa00750	Vitamin B6 metabolism	0.997735
2	161	path:hsa00072	Synthesis and degradation of ketone bodies	0.940425
3	92	path:hsa04122	Sulfur relay system	0.855753
4	95	path:hsa00400	Phenylalanine, tyrosine and tryptophan biosynthesis	0.850563
5	277	path:hsa00533	Glycosaminoglycan biosynthesis - keratan sulfate	0.836469
6	165	path:hsa04964	Proximal tubule bicarbonate reclamation	0.803311
7	110	path:hsa01040	Biosynthesis of unsaturated fatty acids	0.799334
8	262	path:hsa00630	Glyoxylate and dicarboxylate metabolism	0.785954
9	146	path:hsa05217	Basal cell carcinoma	0.779876
10	227	path:hsa00910	Nitrogen metabolism	0.77962
11	55	path:hsa05218	Melanoma	0.758975
12	192	path:hsa04972	Pancreatic secretion	0.754263
13	129	path:hsa00670	One carbon pool by folate	0.7452
14	184	path:hsa00900	Terpenoid backbone biosynthesis	0.736641
15	263	path:hsa00920	Sulfur metabolism	0.733627
16	170	path:hsa00260	Glycine, serine and threonine metabolism	0.722685
17	194	path:hsa00062	Fatty acid elongation	0.721316
18	173	path:hsa00982	Drug metabolism - cytochrome P450	0.719736
19	6	path:hsa04320	Dorso-ventral axis formation	0.716266
20	149	path:hsa05214	Glioma	0.715543
21	158	path:hsa00071	Fatty acid degradation	0.714148
22	190	path:hsa04970	Salivary secretion	0.712809
23	154	path:hsa03020	RNA polymerase	0.708837
24	60	path:hsa05219	Bladder cancer	0.707041
25	252	path:hsa05216	Thyroid cancer	0.705947
26	71	path:hsa04614	Renin-angiotensin system	0.704802
27	135	path:hsa00512	Mucin type O-Glycan biosynthesis	0.704588
28	128	path:hsa04130	SNARE interactions in vesicular transport	0.704397
29	89	path:hsa04976	Bile secretion	0.702732
30	214	path:hsa05204	Chemical carcinogenesis	0.700821
31	280	path:hsa04340	Hedgehog signaling pathway	0.700107
32	182	path:hsa00591	Linoleic acid metabolism	0.6986
33	169	path:hsa04270	Vascular smooth muscle contraction	0.694864
34	7	path:hsa00380	Tryptophan metabolism	0.687638
35	145	path:hsa02010	ABC transporters	0.686661
36	160	path:hsa04020	Calcium signaling pathway	0.68589
37	203	path:hsa04260	Cardiac muscle contraction	0.685434
38	207	path:hsa00230	Purine metabolism	0.685324
39	54	path:hsa04520	Adherens junction	0.683596
40	197	path:hsa00061	Fatty acid biosynthesis	0.682065
41	102	path:hsa04140	Regulation of autophagy	0.681647
42	19	path:hsa00620	Pyruvate metabolism	0.681196
43	43	path:hsa00565	Ether lipid metabolism	0.679973
44	106	path:hsa04973	Carbohydrate digestion and absorption	0.679229

Continued

45	109	path:hsa00460	Cyanoamino acid metabolism	0.678849
46	198	path:hsa04978	Mineral absorption	0.677849
47	140	path:hsa00100	Steroid biosynthesis	0.675419
48	31	path:hsa00330	Arginine and proline metabolism	0.673097
49	269	path:hsa04911	Insulin secretion	0.672386
50	36	path:hsa00010	Glycolysis / Gluconeogenesis	0.672222
51	74	path:hsa04144	Endocytosis	0.670588
52	155	path:hsa04390	Hippo signaling pathway	0.669141
53	63	path:hsa00140	Steroid hormone biosynthesis	0.666393
54	233	path:hsa00280	Valine, leucine and isoleucine degradation	0.666238
55	10	path:hsa04750	Inflammatory mediator regulation of TRP channels	0.666109
56	73	path:hsa05412	Arrhythmogenic right ventricular cardiomyopathy (ARVC)	0.666034
57	85	path:hsa00480	Glutathione metabolism	0.66584
58	3	path:hsa00020	Citrate cycle (TCA cycle)	0.665614
59	174	path:hsa04961	Endocrine and other factor-regulated calcium reabsorption	0.665599
60	256	path:hsa05213	Endometrial cancer	0.664796
61	66	path:hsa00410	beta-Alanine metabolism	0.664263
62	80	path:hsa04310	Wnt signaling pathway	0.663738
63	82	path:hsa05221	Acute myeloid leukemia	0.663554
64	205	path:hsa00232	Caffeine metabolism	0.663377
65	52	path:hsa04726	Serotonergic synapse	0.660993
66	267	path:hsa04913	Ovarian steroidogenesis	0.660794
67	204	path:hsa04070	Phosphatidylinositol signaling system	0.658992
68	270	path:hsa04916	Melanogenesis	0.658276
69	23	path:hsa00830	Retinol metabolism	0.657137
70	219	path:hsa05202	Transcriptional misregulation in cancer	0.65703
71	15	path:hsa05220	Chronic myeloid leukemia	0.656977
72	171	path:hsa00980	Metabolism of xenobiotics by cytochrome P450	0.656215
73	181	path:hsa00590	Arachidonic acid metabolism	0.656001
74	278	path:hsa00534	Glycosaminoglycan biosynthesis - heparan sulfate / heparin	0.655741
75	79	path:hsa04530	Tight junction	0.654656
76	189	path:hsa04014	Ras signaling pathway	0.653737
77	24	path:hsa04921	Oxytocin signaling pathway	0.653629
78	247	path:hsa00780	Biotin metabolism	0.652587
79	151	path:hsa05215	Prostate cancer	0.652499
80	45	path:hsa04721	Synaptic vesicle cycle	0.651556
81	185	path:hsa04966	Collecting duct acid secretion	0.649391
82	88	path:hsa04912	GnRH signaling pathway	0.64929
83	136	path:hsa04360	Axon guidance	0.648474
84	111	path:hsa04540	Gap junction	0.646717
85	168	path:hsa04915	Estrogen signaling pathway	0.646177
86	244	path:hsa04060	Cytokine-cytokine receptor interaction	0.645991
87	229	path:hsa04080	Neuroactive ligand-receptor interaction	0.644434
88	12	path:hsa05223	Non-small cell lung cancer	0.643901
89	261	path:hsa04919	Thyroid hormone signaling pathway	0.643654

Continued

90	237	path:hsa00350	Tyrosine metabolism	0.641369
91	61	path:hsa00650	Butanoate metabolism	0.64014
92	213	path:hsa05205	Proteoglycans in cancer	0.639961
93	200	path:hsa05145	Toxoplasmosis	0.639809
94	238	path:hsa04022	cGMP-PKG signaling pathway	0.63899
95	178	path:hsa04960	Aldosterone-regulated sodium reabsorption	0.638794
96	216	path:hsa05206	MicroRNAs in cancer	0.638678
97	217	path:hsa05200	Pathways in cancer	0.638114
98	40	path:hsa00563	Glycosylphosphatidylinositol(GPI)-anchor biosynthesis	0.636153
99	164	path:hsa04622	RIG-I-like receptor signaling pathway	0.635685
100	49	path:hsa04725	Cholinergic synapse	0.634438
101	114	path:hsa04971	Gastric acid secretion	0.634435
102	51	path:hsa04727	GABAergic synapse	0.633573
103	21	path:hsa04151	PI3K-Akt signaling pathway	0.63213
104	35	path:hsa00240	Pyrimidine metabolism	0.631842
105	97	path:hsa04012	ErbB signaling pathway	0.631436
106	258	path:hsa04744	Phototransduction	0.631374
107	44	path:hsa00564	Glycerophospholipid metabolism	0.631313
108	225	path:hsa00601	Glycosphingolipid biosynthesis - lacto and neolacto series	0.631216
109	242	path:hsa04062	Chemokine signaling pathway	0.629907
110	30	path:hsa04350	TGF-beta signaling pathway	0.629755
111	99	path:hsa00500	Starch and sucrose metabolism	0.629547
112	17	path:hsa04510	Focal adhesion	0.62937
113	150	path:hsa04261	Adrenergic signaling in cardiomyocytes	0.629021
114	67	path:hsa05414	Dilated cardiomyopathy	0.628971
115	14	path:hsa04514	Cell adhesion molecules (CAMs)	0.628512
116	193	path:hsa04010	MAPK signaling pathway	0.627698
117	148	path:hsa05410	Hypertrophic cardiomyopathy (HCM)	0.627539
118	172	path:hsa00983	Drug metabolism - other enzymes	0.627089
119	239	path:hsa00520	Amino sugar and nucleotide sugar metabolism	0.627068
120	37	path:hsa00051	Fructose and mannose metabolism	0.62685
121	121	path:hsa04713	Circadian entrainment	0.626742
122	100	path:hsa04146	Peroxisome	0.626588
123	70	path:hsa04630	Jak-STAT signaling pathway	0.625882
124	147	path:hsa00970	Aminoacyl-tRNA biosynthesis	0.625169
125	119	path:hsa03018	RNA degradation	0.625114
126	138	path:hsa00340	Histidine metabolism	0.624957
127	188	path:hsa04015	Rap1 signaling pathway	0.624508
128	215	path:hsa04917	Prolactin signaling pathway	0.62365
129	271	path:hsa04672	Intestinal immune network for IgA production	0.621944
130	41	path:hsa00562	Inositol phosphate metabolism	0.621914
131	260	path:hsa03050	Proteasome	0.621785
132	180	path:hsa00592	alpha-Linolenic acid metabolism	0.621505
133	253	path:hsa04742	Taste transduction	0.620231
134	127	path:hsa04810	Regulation of actin cytoskeleton	0.61998
135	132	path:hsa05120	Epithelial cell signaling in Helicobacter pylori infection	0.619948
136	254	path:hsa04740	Olfactory transduction	0.619626

Continued

137	232	path:hsa00052	Galactose metabolism	0.619562
138	220	path:hsa05160	Hepatitis C	0.617832
139	38	path:hsa05211	Renal cell carcinoma	0.61744
140	142	path:hsa03420	Nucleotide excision repair	0.6174
141	94	path:hsa04120	Ubiquitin mediated proteolysis	0.616345
142	133	path:hsa04950	Maturity onset diabetes of the young	0.61574
143	22	path:hsa00511	Other glycan degradation	0.615321
144	276	path:hsa00532	Glycosaminoglycan biosynthesis - chondroitin sulfate / dermatan sulfate	0.614719
145	240	path:hsa04210	Apoptosis	0.613644
146	201	path:hsa00430	Taurine and hypotaurine metabolism	0.613338
147	231	path:hsa00053	Ascorbate and aldarate metabolism	0.612301
148	230	path:hsa04380	Osteoclast differentiation	0.612047
149	255	path:hsa05212	Pancreatic cancer	0.611774
150	139	path:hsa04152	AMPK signaling pathway	0.61031
151	46	path:hsa04720	Long-term potentiation	0.609893
152	48	path:hsa04722	Neurotrophin signaling pathway	0.609835
153	47	path:hsa04723	Retrograde endocannabinoid signaling	0.609795
154	251	path:hsa05169	Epstein-Barr virus infection	0.60962
155	104	path:hsa05010	Alzheimer,s disease	0.608975
156	75	path:hsa04918	Thyroid hormone synthesis	0.608098
157	50	path:hsa04724	Glutamatergic synapse	0.60772
158	226	path:hsa05162	Measles	0.60755
159	87	path:hsa00760	Nicotinate and nicotinamide metabolism	0.607489
160	108	path:hsa04962	Vasopressin-regulated water reabsorption	0.606223
161	90	path:hsa00310	Lysine degradation	0.605012
162	120	path:hsa04068	FoxO signaling pathway	0.604155
163	112	path:hsa05016	Huntington,s disease	0.603048
164	69	path:hsa05416	Viral myocarditis	0.602887
165	143	path:hsa04710	Circadian rhythm	0.600736
166	234	path:hsa05164	Influenza A	0.600596
167	72	path:hsa00360	Phenylalanine metabolism	0.599565
168	115	path:hsa04668	TNF signaling pathway	0.596904
169	57	path:hsa05332	Graft-versus-host disease	0.596546
170	273	path:hsa04670	Leukocyte transendothelial migration	0.594611
171	86	path:hsa03008	Ribosome biogenesis in eukaryotes	0.594596
172	68	path:hsa04066	HIF-1 signaling pathway	0.594228
173	259	path:hsa05032	Morphine addiction	0.594205
174	76	path:hsa00471	D-Glutamine and D-glutamate metabolism	0.592886
175	224	path:hsa00600	Sphingolipid metabolism	0.592886
176	156	path:hsa05110	Vibrio cholerae infection	0.592755
177	53	path:hsa04728	Dopaminergic synapse	0.592747
178	195	path:hsa04650	Natural killer cell mediated cytotoxicity	0.591723
179	274	path:hsa05168	Herpes simplex infection	0.591686
180	163	path:hsa04621	NOD-like receptor signaling pathway	0.590824
181	206	path:hsa05100	Bacterial invasion of epithelial cells	0.590789
182	78	path:hsa04730	Long-term depression	0.589558

Continued

183	125	path:hsa03013	RNA transport	0.589412
184	42	path:hsa00561	Glycerolipid metabolism	0.587395
185	175	path:hsa00190	Oxidative phosphorylation	0.587331
186	101	path:hsa04141	Protein processing in endoplasmic reticulum	0.587077
187	218	path:hsa05203	Viral carcinogenesis	0.58683
188	56	path:hsa05330	Allograft rejection	0.585975
189	26	path:hsa04660	T cell receptor signaling pathway	0.582007
190	266	path:hsa00040	Pentose and glucuronate interconversions	0.578792
191	211	path:hsa05130	Pathogenic Escherichia coli infection	0.578558
192	107	path:hsa05014	Amyotrophic lateral sclerosis (ALS)	0.578478
193	177	path:hsa00860	Porphyrin and chlorophyll metabolism	0.576713
194	222	path:hsa05012	Parkinson,s disease	0.575914
195	235	path:hsa04640	Hematopoietic cell lineage	0.574836
196	272	path:hsa00730	Thiamine metabolism	0.574597
197	130	path:hsa04623	Cytosolic DNA-sensing pathway	0.57417
198	118	path:hsa05166	HTLV-I infection	0.574079
199	210	path:hsa05031	Amphetamine addiction	0.573941
200	93	path:hsa00640	Propanoate metabolism	0.57385
201	32	path:hsa00130	Ubiquinone and other terpenoid-quinone biosynthesis	0.573767
202	103	path:hsa04142	Lysosome	0.572043
203	268	path:hsa04910	Insulin signaling pathway	0.571738
204	228	path:hsa05323	Rheumatoid arthritis	0.571427
205	134	path:hsa00270	Cysteine and methionine metabolism	0.571371
206	208	path:hsa03320	PPAR signaling pathway	0.571139
207	59	path:hsa04932	Non-alcoholic fatty liver disease (NAFLD)	0.570612
208	187	path:hsa04975	Fat digestion and absorption	0.568677
209	157	path:hsa05142	Chagas disease (American trypanosomiasis)	0.568202
210	223	path:hsa00603	Glycosphingolipid biosynthesis - globo series	0.568034
211	84	path:hsa05321	Inflammatory bowel disease (IBD)	0.568026
212	137	path:hsa00510	N-Glycan biosynthesis	0.566892
213	18	path:hsa03060	Protein export	0.566703
214	250	path:hsa03450	Non-homologous end-joining	0.564347
215	221	path:hsa05161	Hepatitis B	0.564038
216	4	path:hsa04612	Antigen processing and presentation	0.563928
217	257	path:hsa05210	Colorectal cancer	0.563923
218	141	path:hsa00514	Other types of O-glycan biosynthesis	0.562149
219	183	path:hsa05152	Tuberculosis	0.560461
220	264	path:hsa05034	Alcoholism	0.558711
221	16	path:hsa04512	ECM-receptor interaction	0.557914
222	117	path:hsa04611	Platelet activation	0.556926
223	245	path:hsa03040	Spliceosome	0.556297
224	83	path:hsa05320	Autoimmune thyroid disease	0.555865
225	167	path:hsa04370	VEGF signaling pathway	0.552802
226	241	path:hsa04064	NF-kappa B signaling pathway	0.552006
227	144	path:hsa00450	Selenocompound metabolism	0.550685
228	153	path:hsa03022	Basal transcription factors	0.549934
229	98	path:hsa04145	Phagosome	0.548957

Continued

230	166	path:hsa03430	Mismatch repair	0.548792
231	11	path:hsa00604	Glycosphingolipid biosynthesis - ganglio series	0.548246
232	58	path:hsa04930	Type II diabetes mellitus	0.547685
233	162	path:hsa04620	Toll-like receptor signaling pathway	0.547334
234	13	path:hsa05222	Small cell lung cancer	0.5461
235	122	path:hsa05146	Amoebiasis	0.545818
236	124	path:hsa03015	mRNA surveillance pathway	0.54414
237	116	path:hsa05310	Asthma	0.539444
238	152	path:hsa00740	Riboflavin metabolism	0.538229
239	25	path:hsa04920	Adipocytokine signaling pathway	0.536513
240	2	path:hsa04610	Complement and coagulation cascades	0.535967
241	81	path:hsa05322	Systemic lupus erythematosus	0.530779
242	236	path:hsa00524	Butirosin and neomycin biosynthesis	0.529456
243	91	path:hsa04977	Vitamin digestion and absorption	0.529347
244	28	path:hsa04664	Fc epsilon RI signaling pathway	0.527913
245	248	path:hsa00785	Lipoic acid metabolism	0.52177
246	243	path:hsa00030	Pentose phosphate pathway	0.521448
247	34	path:hsa05132	Salmonella infection	0.520561
248	196	path:hsa05140	Leishmaniasis	0.518409
249	249	path:hsa04330	Notch signaling pathway	0.518105
250	1	path:hsa00790	Folate biosynthesis	0.51757
251	96	path:hsa04940	Type I diabetes mellitus	0.514857
252	209	path:hsa05131	Shigellosis	0.514325
253	179	path:hsa03030	DNA replication	0.513507
254	27	path:hsa04662	B cell receptor signaling pathway	0.511445
255	9	path:hsa03460	Fanconi anemia pathway	0.508786
256	202	path:hsa00120	Primary bile acid biosynthesis	0.501413
257	105	path:hsa03410	Base excision repair	0.498963
258	5	path:hsa05340	Primary immunodeficiency	0.498545
259	39	path:hsa05134	Legionellosis	0.498354
260	246	path:hsa05033	Nicotine addiction	0.494798
261	33	path:hsa05133	Pertussis	0.491721
262	199	path:hsa05144	Malaria	0.488162
263	123	path:hsa00770	Pantothenate and CoA biosynthesis	0.486534
264	159	path:hsa05150	Staphylococcus aureus infection	0.484692
265	8	path:hsa05020	Prion diseases	0.480974
266	65	path:hsa04114	Oocyte meiosis	0.480951
267	186	path:hsa04974	Protein digestion and absorption	0.477538
268	29	path:hsa04666	Fc gamma R-mediated phagocytosis	0.477385
269	131	path:hsa04115	p53 signaling pathway	0.473868
270	265	path:hsa05030	Cocaine addiction	0.462308
271	279	path:hsa04914	Progesterone-mediated oocyte maturation	0.456377
272	113	path:hsa05143	African trypanosomiasis	0.456038
273	20	path:hsa04150	mTOR signaling pathway	0.453409
274	191	path:hsa00290	Valine, leucine and isoleucine biosynthesis	0.446511
275	64	path:hsa04110	Cell cycle	0.446004
276	212	path:hsa03440	Homologous recombination	0.441897

Continued

277	275	path:hsa00531	Glycosaminoglycan degradation	0.439597
278	126	path:hsa03010	Ribosome	0.300678
279	62	path:hsa00300	Lysine biosynthesis	0.236189
280	77	path:hsa00472	D-Arginine and D-ornithine metabolism	NA
