# Supplementary material for: A network-based pathway-expanding approach for pathway analysis
Source: BMC Bioinformatics. 2016 Dec 23;17(Suppl 17):536. doi: 10.1186/s12859-016-1333-x (PMC5259956; doi:10.1186/s12859-016-1333-x)
Supplement: Additional file 2 — Table S2. The results of GSEA from BRCA. (PDF 321 kb) [file 12859_2016_1333_MOESM2_ESM.pdf]

Table S2. The results of GSEA from BRCA

Rank	Entry	Name	NES	NOM p-val	FDR q-val	FWER p-val
1	path:hsa05322	Systemic lupus erythematosus	2.735978	0	0	0
2	path:hsa00982	Drug metabolism - cytochrome P450	2.460232	0	0	0
3	path:hsa05204	Chemical carcinogenesis	2.337143	0	0	0
4	path:hsa03030	DNA replication	2.267892	0	0	0
5	path:hsa04110	Cell cycle	2.226303	0	0	0
6	path:hsa00980	Metabolism of xenobiotics by cytochrome P450	2.223599	0	8.14E-04	0.002
7	path:hsa05034	Alcoholism	2.177176	0	0	0
8	path:hsa03460	Fanconi anemia pathway	2.167901	0	0	0
9	path:hsa05203	Viral carcinogenesis	2.158631	0	0	0
10	path:hsa00071	Fatty acid degradation	2.156569	0	6.10E-04	0.002
11	path:hsa03050	Proteasome	2.107461	0	1.16E-04	0.001
12	path:hsa00830	Retinol metabolism	2.104021	0	0.001421	0.006
13	path:hsa03410	Base excision repair	2.073891	0	1.01E-04	0.001
14	path:hsa03320	PPAR signaling pathway	1.993659	0	0.003776	0.019
15	path:hsa00240	Pyrimidine metabolism	1.970454	0	9.46E-04	0.01
16	path:hsa03010	Ribosome	1.953796	0	0.00518	0.03
17	path:hsa04142	Lysosome	1.92517	0	0.002105	0.024
18	path:hsa03440	Homologous recombination	1.924983	0.0018587	0.001914	0.024
19	path:hsa00100	Steroid biosynthesis	1.8914	0	0.002805	0.039
20	path:hsa00280	Valine, leucine and isoleucine	1.89088	0	0.008925	0.059
21	path:hsa00640	Propanoate metabolism	1.884771	0.0019763	0.008052	0.06
22	path:hsa04610	Complement and coagulation	1.876063	0	0.008212	0.066
23	path:hsa04141	Protein processing in endoplasmic	1.872864	0	0.003571	0.054
24	path:hsa00970	Aminoacyl-tRNA biosynthesis	1.871583	0	0.003316	0.054
25	path:hsa03040	Spliceosome	1.85548	0	0.003671	0.063
26	path:hsa03430	Mismatch repair	1.852779	0.0018727	0.003661	0.066
27	path:hsa00350	Tyrosine metabolism	1.838361	0	0.011347	0.099
28	path:hsa00510	N-Glycan biosynthesis	1.8117	0	0.005777	0.104
29	path:hsa04060	Cytokine-cytokine receptor	1.733674	0	0.034477	0.291
30	path:hsa04964	Proximal tubule bicarbonate	1.715761	0.0063966	0.038174	0.338
31	path:hsa04612	Antigen processing and presentation	1.697929	0.0018349	0.022338	0.373
32	path:hsa04022	cGMP-PKG signaling pathway	1.696707	0	0.042842	0.393
33	path:hsa04721	Synaptic vesicle cycle	1.695934	0.0018083	0.021738	0.385
34	path:hsa00053	Ascorbate and aldarate metabolism	1.674221	0.0173913	0.049919	0.472
35	path:hsa00410	beta-Alanine metabolism	1.667569	0.0154525	0.049742	0.497
36	path:hsa05110	Vibrio cholerae infection	1.664165	0.0035211	0.028985	0.495
37	path:hsa00650	Butanoate metabolism	1.659663	0.0061856	0.051103	0.532
38	path:hsa04913	Ovarian steroidogenesis	1.645668	0.0088106	0.054897	0.58
39	path:hsa00590	Arachidonic acid metabolism	1.635775	0.0043668	0.056565	0.614
40	path:hsa04114	Oocyte meiosis	1.629066	0.0017699	0.037757	0.615
41	path:hsa05169	Epstein-Barr virus infection	1.62333	0	0.038111	0.631
42	path:hsa04270	Vascular smooth muscle contraction	1.606724	0.002331	0.069704	0.714
43	path:hsa00140	Steroid hormone biosynthesis	1.600013	0.0042105	0.070481	0.736
44	path:hsa03008	Ribosome biogenesis in eukaryotes	1.593007	0.0019685	0.04904	0.753
45	path:hsa03015	mRNA surveillance pathway	1.588761	0.0056285	0.048608	0.765
46	path:hsa03013	RNA transport	1.581327	0.0018832	0.050648	0.786
47	path:hsa00340	Histidine metabolism	1.574917	0.0238612	0.083427	0.799
48	path:hsa00380	Tryptophan metabolism	1.557319	0.0065076	0.091763	0.836
49	path:hsa04623	Cytosolic DNA-sensing pathway	1.546583	0.0182482	0.066341	0.883
50	path:hsa00051	Fructose and mannose metabolism	1.537962	0.0194004	0.068477	0.901
51	path:hsa04360	Axon guidance	1.523003	0	0.115191	0.9
52	path:hsa00910	Nitrogen metabolism	1.522651	0.0479452	0.110814	0.9

Continued

53	path:hsa00360	Phenylalanine metabolism	1.518623	0.0541126	0.110221	0.908
54	path:hsa05031	Amphetamine addiction	1.515144	0.0196078	0.109017	0.914
55	path:hsa03420	Nucleotide excision repair	1.510371	0.0240296	0.083918	0.952
56	path:hsa00010	Glycolysis / Gluconeogenesis	1.503006	0.020316	0.115772	0.935
57	path:hsa05219	Bladder cancer	1.498929	0.0351852	0.089827	0.962
58	path:hsa05131	Shigellosis	1.494669	0.0235081	0.089946	0.965
59	path:hsa04510	Focal adhesion	1.49452	0	0.118853	0.948
60	path:hsa00561	Glycerolipid metabolism	1.485757	0.0293454	0.122671	0.96
61	path:hsa00072	Synthesis and degradation of ketone	1.482445	0.0770833	0.121848	0.96
62	path:hsa04014	Ras signaling pathway	1.478641	0.0048309	0.121982	0.965
63	path:hsa04152	AMPK signaling pathway	1.476273	0.0045767	0.120535	0.968
64	path:hsa00565	Ether lipid metabolism	1.472922	0.0390456	0.120083	0.972
65	path:hsa03020	RNA polymerase	1.472553	0.0479574	0.105033	0.984
66	path:hsa02010	ABC transporters	1.471957	0.0367347	0.117571	0.973
67	path:hsa05164	Influenza A	1.45948	0.0050676	0.113291	0.989
68	path:hsa00500	Starch and sucrose metabolism	1.4588	0.0199557	0.126697	0.983
69	path:hsa04920	Adipocytokine signaling pathway	1.455003	0.0239651	0.127565	0.985
70	path:hsa04914	Progesterone-mediated oocyte	1.454983	0.0229277	0.114012	0.991
71	path:hsa00630	Glyoxylate and dicarboxylate	1.453637	0.0571429	0.1253	0.985
72	path:hsa04640	Hematopoietic cell lineage	1.452845	0.0202703	0.122717	0.986
73	path:hsa00520	Amino sugar and nucleotide sugar metabolism	1.452097	0.0335196	0.113295	0.992
74	path:hsa00260	Glycine, serine and threonine	1.449024	0.0297483	0.123703	0.989
75	path:hsa05020	Prion diseases	1.445381	0.0453564	0.12437	0.989
76	path:hsa00190	Oxidative phosphorylation	1.445244	0.0163934	0.116753	0.994
77	path:hsa05205	Proteoglycans in cancer	1.444212	0	0.122406	0.99
78	path:hsa05032	Morphine addiction	1.443791	0.0160183	0.119955	0.99
79	path:hsa04015	Rap1 signaling pathway	1.440916	0.0050505	0.119662	0.991
80	path:hsa04080	Neuroactive ligand-receptor	1.437723	0	0.1202	0.993
81	path:hsa00020	Citrate cycle (TCA cycle)	1.437107	0.0513393	0.118122	0.993
82	path:hsa00785	Lipoic acid metabolism	1.431054	0.0327198	0.120345	0.997
83	path:hsa04921	Oxytocin signaling pathway	1.430823	0.0072464	0.117987	0.997
84	path:hsa04020	Calcium signaling pathway	1.418657	0.0091324	0.126322	0.998
85	path:hsa04978	Mineral absorption	1.412759	0.0277186	0.128448	0.999
86	path:hsa04614	Renin-angiotensin system	1.3982	0.0820734	0.138631	0.999
87	path:hsa00511	Other glycan degradation	1.397368	0.0885827	0.16259	0.999
88	path:hsa05152	Tuberculosis	1.394621	0.019678	0.161751	0.999
89	path:hsa00620	Pyruvate metabolism	1.380778	0.0580357	0.152891	1
90	path:hsa04630	Jak-STAT signaling pathway	1.375733	0.015625	0.15562	1
91	path:hsa04151	PI3K-Akt signaling pathway	1.375246	0	0.153205	1
92	path:hsa05218	Melanoma	1.368143	0.0532407	0.158315	1
93	path:hsa04713	Circadian entrainment	1.36801	0.0488372	0.155546	1
94	path:hsa05016	Huntington,s disease	1.366691	0.0179533	0.193197	1
95	path:hsa00524	Butirosin and neomycin biosynthesis	1.363828	0.0970297	0.192102	1
96	path:hsa04145	Phagosome	1.36248	0.0249554	0.189032	1
97	path:hsa00534	Glycosaminoglycan biosynthesis - heparan sulfate / heparin	1.358561	0.0887574	0.189909	1
98	path:hsa04350	TGF-beta signaling pathway	1.356619	0.0544662	0.164873	1
99	path:hsa00040	Pentose and glucuronate	1.352845	0.1073684	0.166375	1
100	path:hsa04261	Adrenergic signaling in	1.35106	0.0236967	0.165542	1
101	path:hsa00230	Purine metabolism	1.343978	0.0176367	0.205782	1
102	path:hsa03450	Non-homologous end-joining	1.339056	0.12939	0.207686	1
103	path:hsa00430	Taurine and hypotaurine	1.335183	0.1445783	0.180799	1
104	path:hsa04966	Collecting duct acid secretion	1.333774	0.1086957	0.210611	1
105	path:hsa05212	Pancreatic cancer	1.333266	0.0491493	0.206607	1
106	path:hsa03060	Protein export	1.327351	0.1123389	0.21087	1

Continued

107	path:hsa04120	Ubiquitin mediated proteolysis	1.322194	0.0242634	0.213654	1
108	path:hsa03018	RNA degradation	1.317816	0.0530973	0.21536	1
109	path:hsa04972	Pancreatic secretion	1.315877	0.0772834	0.200955	1
110	path:hsa05161	Hepatitis B	1.310903	0.0419708	0.221729	1
111	path:hsa00563	Glycosylphosphatidylinositol(GPI)-anchor biosynthesis	1.302244	0.1281071	0.230611	1
112	path:hsa00270	Cysteine and methionine	1.293028	0.1007194	0.240358	1
113	path:hsa00603	Glycosphingolipid biosynthesis - globo series	1.29277	0.1609196	0.236311	1
114	path:hsa00290	Valine, leucine and isoleucine	1.288579	0.1567878	0.238426	1
115	path:hsa05144	Malaria	1.286369	0.1208054	0.238935	1
116	path:hsa05130	Pathogenic Escherichia coli infection	1.276562	0.1175373	0.253918	1
117	path:hsa04724	Glutamatergic synapse	1.274573	0.062635	0.252494	1
118	path:hsa05160	Hepatitis C	1.270675	0.0606061	0.259202	1
119	path:hsa04730	Long-term depression	1.268819	0.1235294	0.258054	1
120	path:hsa04971	Gastric acid secretion	1.268327	0.0988764	0.254682	1
121	path:hsa04320	Dorso-ventral axis formation	1.267659	0.1666667	0.252098	1
122	path:hsa00533	Glycosaminoglycan biosynthesis - keratan sulfate	1.262178	0.1897533	0.268085	1
123	path:hsa05217	Basal cell carcinoma	1.252819	0.129979	0.27122	1
124	path:hsa04662	B cell receptor signaling pathway	1.251552	0.1203704	0.282394	1
125	path:hsa04530	Tight junction	1.246977	0.0406321	0.276418	1
126	path:hsa05168	Herpes simplex infection	1.245445	0.0850695	0.289018	1
127	path:hsa04976	Bile secretion	1.239644	0.1054945	0.284649	1
128	path:hsa04728	Dopaminergic synapse	1.238088	0.0774488	0.283448	1
129	path:hsa05162	Measles	1.235156	0.1058201	0.302678	1
130	path:hsa00400	Phenylalanine, tyrosine and tryptophan biosynthesis	1.223038	0.2480916	0.320771	1
131	path:hsa04068	FoxO signaling pathway	1.220495	0.0796253	0.308394	1
132	path:hsa04010	MAPK signaling pathway	1.217782	0.0559284	0.308958	1
133	path:hsa05133	Pertussis	1.215467	0.1503268	0.308409	1
134	path:hsa05340	Primary immunodeficiency	1.212584	0.1719368	0.335806	1
135	path:hsa04750	Inflammatory mediator regulation of TRP channels	1.212449	0.1308411	0.309697	1
136	path:hsa05206	MicroRNAs in cancer	1.200974	0.1003521	0.353069	1
137	path:hsa00591	Linoleic acid metabolism	1.197585	0.1973094	0.332544	1
138	path:hsa04115	p53 signaling pathway	1.186375	0.1761566	0.377569	1
139	path:hsa04064	NF-kappa B signaling pathway	1.184192	0.145359	0.376175	1
140	path:hsa04622	RIG-I-like receptor signaling pathway	1.183719	0.1776557	0.371381	1
141	path:hsa05410	Hypertrophic cardiomyopathy	1.183243	0.1407249	0.355654	1
142	path:hsa05010	Alzheimer,s disease	1.176847	0.1153184	0.37949	1
143	path:hsa05414	Dilated cardiomyopathy	1.174365	0.14862	0.369078	1
144	path:hsa05202	Transcriptional misregulation in	1.172912	0.1090047	0.367244	1
145	path:hsa04620	Toll-like receptor signaling pathway	1.16668	0.1763636	0.395582	1
146	path:hsa05220	Chronic myeloid leukemia	1.166286	0.1954625	0.390577	1
147	path:hsa05200	Pathways in cancer	1.166016	0.0767386	0.376646	1
148	path:hsa05323	Rheumatoid arthritis	1.162233	0.1989437	0.393809	1
149	path:hsa00512	Mucin type O-Glycan biosynthesis	1.161849	0.2450451	0.38915	1
150	path:hsa04150	mTOR signaling pathway	1.158096	0.2017544	0.38803	1
151	path:hsa04512	ECM-receptor interaction	1.156737	0.1990741	0.385969	1
152	path:hsa00062	Fatty acid elongation	1.156139	0.258547	0.382505	1
153	path:hsa04390	Hippo signaling pathway	1.154138	0.1394336	0.38198	1
154	path:hsa00740	Riboflavin metabolism	1.151185	0.2912622	0.406905	1
155	path:hsa04726	Serotonergic synapse	1.15043	0.2067416	0.385332	1
156	path:hsa04723	Retrograde endocannabinoid	1.149599	0.1682692	0.382522	1

Continued

157	path:hsa04668	TNF signaling pathway	1.14575	0.1841492	0.386005	1
158	path:hsa04960	Aldosterone-regulated sodium	1.144147	0.2454955	0.384633	1
159	path:hsa04146	Peroxisome	1.139159	0.2044944	0.390441	1
160	path:hsa00330	Arginine and proline metabolism	1.134112	0.2431942	0.439652	1
161	path:hsa00480	Glutathione metabolism	1.133033	0.2594142	0.398584	1
162	path:hsa00564	Glycerophospholipid metabolism	1.129862	0.2403628	0.400442	1
163	path:hsa04710	Circadian rhythm	1.119106	0.2561475	0.419843	1
164	path:hsa05030	Cocaine addiction	1.116604	0.2592593	0.420683	1
165	path:hsa05132	Salmonella infection	1.11512	0.2476636	0.419227	1
166	path:hsa00471	D-Glutamine and D-glutamate	1.114001	0.3306931	0.417363	1
167	path:hsa05216	Thyroid cancer	1.113629	0.2779851	0.481178	1
168	path:hsa00514	Other types of O-glycan biosynthesis	1.110304	0.3068592	0.483116	1
169	path:hsa05143	African trypanosomiasis	1.109247	0.2934537	0.423097	1
170	path:hsa04520	Adherens junction	1.106756	0.2828947	0.42416	1
171	path:hsa05142	Chagas disease (American	1.105934	0.2312925	0.421546	1
172	path:hsa04930	Type II diabetes mellitus	1.098525	0.2980132	0.432849	1
173	path:hsa05166	HTLV-I infection	1.09587	0.2129472	0.51355	1
174	path:hsa04910	Insulin signaling pathway	1.094554	0.2477478	0.437379	1
175	path:hsa04916	Melanogenesis	1.093086	0.2528217	0.436038	1
176	path:hsa04660	T cell receptor signaling pathway	1.088945	0.2814815	0.523941	1
177	path:hsa05213	Endometrial cancer	1.086743	0.3278689	0.522835	1
178	path:hsa05033	Nicotine addiction	1.085291	0.3040541	0.448712	1
179	path:hsa00450	Selenocompound metabolism	1.084636	0.3531598	0.521262	1
180	path:hsa04977	Vitamin digestion and absorption	1.06864	0.3568465	0.482413	1
181	path:hsa00670	One carbon pool by folate	1.064831	0.3652482	0.565192	1
182	path:hsa05211	Renal cell carcinoma	1.064179	0.3282732	0.559853	1
183	path:hsa04720	Long-term potentiation	1.06396	0.3224401	0.488107	1
184	path:hsa00300	Lysine biosynthesis	1.059537	0.4348697	0.493688	1
185	path:hsa04975	Fat digestion and absorption	1.055231	0.3644068	0.499552	1
186	path:hsa00531	Glycosaminoglycan degradation	1.051117	0.3765996	0.587194	1
187	path:hsa03022	Basal transcription factors	1.046617	0.3733826	0.59277	1
188	path:hsa05223	Non-small cell lung cancer	1.043493	0.3674589	0.593557	1
189	path:hsa05140	Leishmaniasis	1.041235	0.3829401	0.592156	1
190	path:hsa00600	Sphingolipid metabolism	1.041048	0.3825243	0.58572	1
191	path:hsa05134	Legionellosis	1.037597	0.3917051	0.538138	1
192	path:hsa00760	Nicotinate and nicotinamide	1.035993	0.4090909	0.537062	1
193	path:hsa05412	Arrhythmogenic right ventricular cardiomyopathy (ARVC)	1.025651	0.3810573	0.558296	1
194	path:hsa04974	Protein digestion and absorption	1.021386	0.3977273	0.563675	1
195	path:hsa04970	Salivary secretion	1.009948	0.4241071	0.587532	1
196	path:hsa05012	Parkinson,s disease	1.008417	0.4303351	0.667359	1
197	path:hsa04540	Gap junction	1.006248	0.4376417	0.591472	1
198	path:hsa04810	Regulation of actin cytoskeleton	1.005918	0.4253898	0.586992	1
199	path:hsa04666	Fc gamma R-mediated phagocytosis	1.000179	0.4480287	0.683797	1
200	path:hsa05215	Prostate cancer	0.998617	0.4584838	0.680574	1
201	path:hsa05221	Acute myeloid leukemia	0.994494	0.4645523	0.684855	1
202	path:hsa00052	Galactose metabolism	0.993054	0.4680851	0.681361	1
203	path:hsa04066	HIF-1 signaling pathway	0.991401	0.478022	0.67827	1
204	path:hsa05100	Bacterial invasion of epithelial cells	0.988163	0.4761905	0.680338	1
205	path:hsa05150	Staphylococcus aureus infection	0.9861	0.4657534	0.63358	1
206	path:hsa04744	Phototransduction	0.983393	0.4557522	0.63543	1
207	path:hsa00900	Terpenoid backbone biosynthesis	0.979538	0.4924528	0.696605	1
208	path:hsa04310	Wnt signaling pathway	0.978239	0.4773756	0.643842	1
209	path:hsa04912	GnRH signaling pathway	0.972543	0.508658	0.653721	1
210	path:hsa00780	Biotin metabolism	0.970013	0.55	0.716227	1
211	path:hsa04919	Thyroid hormone signaling pathway	0.965071	0.5592105	0.668806	1

Continued

212	path:hsa00120	Primary bile acid biosynthesis	0.964568	0.5324675	0.664487	1
213	path:hsa04722	Neurotrophin signaling pathway	0.962263	0.5430712	0.73022	1
214	path:hsa04727	GABAergic synapse	0.959112	0.527027	0.673064	1
215	path:hsa05222	Small cell lung cancer	0.951316	0.5513514	0.744746	1
216	path:hsa04621	NOD-like receptor signaling pathway	0.945895	0.5364807	0.702218	1
217	path:hsa04370	VEGF signaling pathway	0.93916	0.5673759	0.769469	1
218	path:hsa04973	Carbohydrate digestion and	0.935587	0.5578704	0.723186	1
219	path:hsa04514	Cell adhesion molecules (CAMs)	0.922471	0.6566125	0.751547	1
220	path:hsa04611	Platelet activation	0.92212	0.6386404	0.808055	1
221	path:hsa04012	ErbB signaling pathway	0.921674	0.6144579	0.747231	1
222	path:hsa04918	Thyroid hormone synthesis	0.919649	0.6059226	0.74615	1
223	path:hsa05310	Asthma	0.919346	0.5785582	0.807069	1
224	path:hsa05120	Epithelial cell signaling in Helicobacter pylori infection	0.918389	0.6028881	0.801717	1
225	path:hsa00532	Glycosaminoglycan biosynthesis - chondroitin sulfate / dermatan	0.908593	0.5839695	0.820094	1
226	path:hsa05210	Colorectal cancer	0.906462	0.6311927	0.817992	1
227	path:hsa05146	Amoebiasis	0.90574	0.672209	0.775482	1
228	path:hsa04940	Type I diabetes mellitus	0.901254	0.6415441	0.823345	1
229	path:hsa04670	Leukocyte transendothelial	0.900046	0.6913875	0.783687	1
230	path:hsa00061	Fatty acid biosynthesis	0.899851	0.5647321	0.777978	1
231	path:hsa00030	Pentose phosphate pathway	0.891352	0.6208333	0.792704	1
232	path:hsa04917	Prolactin signaling pathway	0.88778	0.6720721	0.849676	1
233	path:hsa04062	Chemokine signaling pathway	0.876402	0.8158784	0.870172	1
234	path:hsa04725	Cholinergic synapse	0.875732	0.7628319	0.863859	1
235	path:hsa00592	alpha-Linolenic acid metabolism	0.875716	0.6577181	0.823815	1
236	path:hsa04915	Estrogen signaling pathway	0.873349	0.7427008	0.861187	1
237	path:hsa00310	Lysine degradation	0.871875	0.6840215	0.85663	1
238	path:hsa05214	Glioma	0.866826	0.7417943	0.839046	1
239	path:hsa04210	Apoptosis	0.860343	0.7433155	0.875556	1
240	path:hsa04380	Osteoclast differentiation	0.854673	0.8357488	0.859126	1
241	path:hsa04260	Cardiac muscle contraction	0.853039	0.7513322	0.884567	1
242	path:hsa04932	Non-alcoholic fatty liver disease	0.851474	0.8407871	0.880177	1
243	path:hsa04911	Insulin secretion	0.850846	0.7792208	0.860484	1
244	path:hsa05014	Amyotrophic lateral sclerosis (ALS)	0.84497	0.781457	0.865791	1
245	path:hsa04144	Endocytosis	0.844214	0.8886986	0.88832	1
246	path:hsa05145	Toxoplasmosis	0.844053	0.8511628	0.861286	1
247	path:hsa00730	Thiamine metabolism	0.844041	0.700565	0.880865	1
248	path:hsa04330	Notch signaling pathway	0.840097	0.7680851	0.863007	1
249	path:hsa04664	Fc epsilon RI signaling pathway	0.837888	0.7533784	0.885648	1
250	path:hsa00460	Cyanoamino acid metabolism	0.836901	0.6884615	0.879909	1
251	path:hsa04130	SNARE interactions in vesicular	0.836389	0.7122302	0.873319	1
252	path:hsa00983	Drug metabolism - other enzymes	0.827653	0.7477273	0.880644	1
253	path:hsa00750	Vitamin B6 metabolism	0.827538	0.6540179	0.874523	1
254	path:hsa00601	Glycosphingolipid biosynthesis - lacto and neolacto series	0.822816	0.7542214	0.891841	1
255	path:hsa04950	Maturity onset diabetes of the	0.799943	0.7851711	0.923897	1
256	path:hsa00770	Pantothenate and CoA biosynthesis	0.79439	0.7374517	0.925065	1
257	path:hsa00472	D-Arginine and D-ornithine	0.791557	0.7992048	0.928474	1
258	path:hsa04961	Endocrine and other factor-regulated calcium reabsorption	0.789114	0.8300654	0.925401	1
259	path:hsa04070	Phosphatidylinositol signaling	0.785282	0.8965517	0.931215	1
260	path:hsa04650	Natural killer cell mediated	0.762392	0.9624574	0.95472	1
261	path:hsa04340	Hedgehog signaling pathway	0.759109	0.8878924	0.95759	1
262	path:hsa04962	Vasopressin-regulated water	0.750827	0.8982684	0.960518	1

Continued

263	path:hsa05416	Viral myocarditis	0.750733	0.9040853	0.960753	1
264	path:hsa00920	Sulfur metabolism	0.743163	0.8285714	0.961118	1
265	path:hsa04672	Intestinal immune network for IgA production	0.72929	0.9225664	0.975281	1
266	path:hsa05330	Allograft rejection	0.72602	0.8782772	0.970387	1
267	path:hsa00562	Inositol phosphate metabolism	0.695249	0.9632224	0.986142	1
268	path:hsa00860	Porphyrin and chlorophyll	0.691041	0.9216758	0.981263	1
269	path:hsa04140	Regulation of autophagy	0.67802	0.9404255	1	1
270	path:hsa04740	Olfactory transduction	0.666322	1	1	1
271	path:hsa00130	Ubiquinone and other terpenoid-quinone biosynthesis	0.664706	0.8777778	0.997914	1
272	path:hsa05321	Inflammatory bowel disease (IBD)	0.647977	0.9848156	0.997921	1
273	path:hsa00604	Glycosphingolipid biosynthesis - ganglio series	0.638395	0.9312639	0.994779	1
274	path:hsa05332	Graft-versus-host disease	0.629413	0.9776952	1	1
275	path:hsa04742	Taste transduction	0.624394	0.9773243	0.992297	1
276	path:hsa04122	Sulfur relay system	0.609889	0.9297913	0.999278	1
277	path:hsa01040	Biosynthesis of unsaturated fatty	0.597239	0.9607477	0.994244	1
278	path:hsa00790	Folate biosynthesis	0.56634	0.9719828	0.998078	1
279	path:hsa00232	Caffeine metabolism	0.502959	0.9723404	0.997311	1
280	path:hsa05320	Autoimmune thyroid disease	0.502891	1	0.997183	1
