# Supplementary material for: A network-based pathway-expanding approach for pathway analysis
Source: BMC Bioinformatics. 2016 Dec 23;17(Suppl 17):536. doi: 10.1186/s12859-016-1333-x (PMC5259956; doi:10.1186/s12859-016-1333-x)
Supplement: Additional file 3 — Table S3. The results of SPIA from BRCA (Top 20). (PDF 110 kb) [file 12859_2016_1333_MOESM3_ESM.pdf]

Table S3. The results of SPIA from BRCA(Top 20)

Rank	Name	Entry	NDE	pNDE	tA	pPERT	pG	pGFdr	pGFWER
1	Focal adhesion	4510	162	0.000372	-105.118	5.00E-06	3.92E-08	5.38E-06	5.38E-06
2	Pathways in cancer	5200	242	0.051756	-128.228	5.00E-06	4.18E-06	0.000287	0.000573
3	Melanoma	5218	52	0.335676	-85.291	5.00E-06	2.40E-05	0.001096	0.003288
4	Gap junction	4540	65	0.169687	-40.775	0.001	0.001643	0.050978	0.225068
5	Cell cycle	4110	104	0.000395	-8.64775	0.497	0.001872	0.050978	0.256471
6	Alcoholism	5034	136	0.000341	6.760131	0.701	0.002233	0.050978	0.305871
7	Tight junction	4530	106	0.000943	1.901832	0.788	0.006096	0.119307	0.835147
8	Small cell lung cancer	5222	65	0.127112	-32.445	0.009	0.008893	0.152286	1
9	Pathogenic Escherichia coli infection	5130	43	0.022993	17.93413	0.068	0.011665	0.177566	1
10	Calcium signaling pathway	4020	116	0.900955	-33.1118	0.002	0.013188	0.180676	1
11	Malaria	5144	42	0.006473	-1.05605	0.51	0.022163	0.257425	1
12	Fanconi anemia pathway	3460	37	0.066082	4.612201	0.053	0.023306	0.257425	1
13	Adipocytokine signaling pathway	4920	54	0.120255	-13.0333	0.031	0.024574	0.257425	1
14	NF-kappa B signaling pathway	4064	69	0.037757	14.89717	0.107	0.026306	0.257425	1
15	Axon guidance	4360	98	0.043059	-15.3606	0.114	0.031007	0.282435	1
16	PPAR signaling pathway	3320	52	0.072375	-3.61671	0.073	0.032985	0.282435	1
17	Glioma	5214	48	0.375277	-34.8254	0.017	0.038627	0.304184	1
18	Prostate cancer	5215	68	0.091037	-25.3771	0.073	0.039966	0.304184	1
19	Neurotrophin signaling pathway	4722	88	0.289706	-30.0009	0.026	0.044355	0.305535	1
20	ErbB signaling pathway	4012	64	0.361111	-40.1278	0.021	0.044604	0.305535	1
