# Supplementary material for: A network-based pathway-expanding approach for pathway analysis
Source: BMC Bioinformatics. 2016 Dec 23;17(Suppl 17):536. doi: 10.1186/s12859-016-1333-x (PMC5259956; doi:10.1186/s12859-016-1333-x)
Supplement: Additional file 4 — Table S4. The scores of pathways from GSE25066. (PDF 272 kb) [file 12859_2016_1333_MOESM4_ESM.pdf]

Table S4. The scores of pathways from GSE25066

Rank	Number	Symbel	Name	Score
1	246	path:hsa05033	Nicotine addiction	0.43065571
2	146	path:hsa05217	Basal cell carcinoma	0.40253392
3	254	path:hsa04740	Olfactory transduction	0.39895226
4	253	path:hsa04742	Taste transduction	0.39306936
5	280	path:hsa04340	Hedgehog signaling pathway	0.37603454
6	51	path:hsa04727	GABAergic synapse	0.36203141
7	121	path:hsa04713	Circadian entrainment	0.35668682
8	231	path:hsa00053	Ascorbate and aldarate metabolism	0.3536296
9	47	path:hsa04723	Retrograde endocannabinoid signaling	0.34331412
10	198	path:hsa04978	Mineral absorption	0.34246095
11	174	path:hsa04961	Endocrine and other factor-regulated calcium reabsorption	0.34161353
12	63	path:hsa00140	Steroid hormone biosynthesis	0.337664
13	185	path:hsa04966	Collecting duct acid secretion	0.33617877
14	249	path:hsa04330	Notch signaling pathway	0.33396368
15	71	path:hsa04614	Renin-angiotensin system	0.33299363
16	89	path:hsa04976	Bile secretion	0.33155797
17	50	path:hsa04724	Glutamatergic synapse	0.33110464
18	258	path:hsa04744	Phototransduction	0.32519225
19	6	path:hsa04320	Dorso-ventral axis formation	0.32464422
20	42	path:hsa00561	Glycerolipid metabolism	0.32354572
21	248	path:hsa00785	Lipoic acid metabolism	0.32290404
22	80	path:hsa04310	Wnt signaling pathway	0.31932572
23	229	path:hsa04080	Neuroactive ligand-receptor interaction	0.31754698
24	269	path:hsa04911	Insulin secretion	0.31488782
25	52	path:hsa04726	Serotonergic synapse	0.31320715
26	105	path:hsa03410	Base excision repair	0.31226416
27	265	path:hsa05030	Cocaine addiction	0.3122355
28	92	path:hsa04122	Sulfur relay system	0.31074156
29	278	path:hsa00534	Glycosaminoglycan biosynthesis - heparan sulfate / heparin	0.30619229
30	94	path:hsa04120	Ubiquitin mediated proteolysis	0.30532663
31	264	path:hsa05034	Alcoholism	0.30463035
32	107	path:hsa05014	Amyotrophic lateral sclerosis (ALS)	0.30452639
33	267	path:hsa04913	Ovarian steroidogenesis	0.30420741
34	25	path:hsa04920	Adipocytokine signaling pathway	0.3040426
35	268	path:hsa04910	Insulin signaling pathway	0.30375578
36	101	path:hsa04141	Protein processing in endoplasmic reticulum	0.30307229
37	236	path:hsa00524	Butirosin and neomycin biosynthesis	0.30280645
38	259	path:hsa05032	Morphine addiction	0.30210387
39	75	path:hsa04918	Thyroid hormone synthesis	0.30165504
40	201	path:hsa00430	Taurine and hypotaurine metabolism	0.30145689
41	238	path:hsa04022	cGMP-PKG signaling pathway	0.301051
42	210	path:hsa05031	Amphetamine addiction	0.30099086
43	143	path:hsa04710	Circadian rhythm	0.30058083
44	203	path:hsa04260	Cardiac muscle contraction	0.300499
45	154	path:hsa03020	RNA polymerase	0.29467213
46	138	path:hsa00340	Histidine metabolism	0.29236256
47	257	path:hsa05210	Colorectal cancer	0.29209495
48	43	path:hsa00565	Ether lipid metabolism	0.29161539
49	45	path:hsa04721	Synaptic vesicle cycle	0.29109733
50	193	path:hsa04010	MAPK signaling pathway	0.29049091
51	261	path:hsa04919	Thyroid hormone signaling pathway	0.28866245
52	270	path:hsa04916	Melanogenesis	0.28756478
53	58	path:hsa04930	Type II diabetes mellitus	0.28750236

Continued

54	233	path:hsa00280	Valine, leucine and isoleucine degradation	0.2862489
55	48	path:hsa04722	Neurotrophin signaling pathway	0.28616311
56	46	path:hsa04720	Long-term potentiation	0.28597289
57	207	path:hsa00230	Purine metabolism	0.28429666
58	99	path:hsa00500	Starch and sucrose metabolism	0.28334615
59	189	path:hsa04014	Ras signaling pathway	0.28241058
60	182	path:hsa00591	Linoleic acid metabolism	0.28218137
61	120	path:hsa04068	FoxO signaling pathway	0.28035766
62	15	path:hsa05220	Chronic myeloid leukemia	0.28015658
63	192	path:hsa04972	Pancreatic secretion	0.28011309
64	108	path:hsa04962	Vasopressin-regulated water reabsorption	0.28002649
65	262	path:hsa00630	Glyoxylate and dicarboxylate metabolism	0.27910643
66	133	path:hsa04950	Maturity onset diabetes of the young	0.27825619
67	252	path:hsa05216	Thyroid cancer	0.27824323
68	190	path:hsa04970	Salivary secretion	0.27615317
69	135	path:hsa00512	Mucin type O-Glycan biosynthesis	0.27547363
70	66	path:hsa00410	beta-Alanine metabolism	0.27456882
71	90	path:hsa00310	Lysine degradation	0.27387607
72	102	path:hsa04140	Regulation of autophagy	0.27360107
73	23	path:hsa00830	Retinol metabolism	0.27341681
74	1	path:hsa00790	Folate biosynthesis	0.27266114
75	202	path:hsa00120	Primary bile acid biosynthesis	0.27072666
76	53	path:hsa04728	Dopaminergic synapse	0.27060741
77	147	path:hsa00970	Aminoacyl-tRNA biosynthesis	0.27055147
78	266	path:hsa00040	Pentose and glucuronate interconversions	0.2703391
79	24	path:hsa04921	Oxytocin signaling pathway	0.26929735
80	20	path:hsa04150	mTOR signaling pathway	0.26889848
81	232	path:hsa00052	Galactose metabolism	0.26884625
82	255	path:hsa05212	Pancreatic cancer	0.26733796
83	150	path:hsa04261	Adrenergic signaling in cardiomyocytes	0.26708527
84	155	path:hsa04390	Hippo signaling pathway	0.26700256
85	112	path:hsa05016	Huntington,s disease	0.26433114
86	36	path:hsa00010	Glycolysis / Gluconeogenesis	0.26358759
87	88	path:hsa04912	GnRH signaling pathway	0.26305826
88	19	path:hsa00620	Pyruvate metabolism	0.2624041
89	256	path:hsa05213	Endometrial cancer	0.26215054
90	177	path:hsa00860	Porphyrin and chlorophyll metabolism	0.26175803
91	100	path:hsa04146	Peroxisome	0.26149969
92	217	path:hsa05200	Pathways in cancer	0.26113309
93	59	path:hsa04932	Non-alcoholic fatty liver disease (NAFLD)	0.25980516
94	106	path:hsa04973	Carbohydrate digestion and absorption	0.2590041
95	151	path:hsa05215	Prostate cancer	0.25818912
96	128	path:hsa04130	SNARE interactions in vesicular transport	0.25810777
97	153	path:hsa03022	Basal transcription factors	0.25797397
98	175	path:hsa00190	Oxidative phosphorylation	0.25793194
99	97	path:hsa04012	ErbB signaling pathway	0.25778009
100	275	path:hsa00531	Glycosaminoglycan degradation	0.25700374
101	49	path:hsa04725	Cholinergic synapse	0.25679006
102	104	path:hsa05010	Alzheimer,s disease	0.25586522
103	37	path:hsa00051	Fructose and mannose metabolism	0.25563628
104	55	path:hsa05218	Melanoma	0.2553623
105	114	path:hsa04971	Gastric acid secretion	0.25526575
106	73	path:hsa05412	Arrhythmogenic right ventricular cardiomyopathy (ARVC)	0.25429262
107	115	path:hsa04668	TNF signaling pathway	0.25368064
108	277	path:hsa00533	Glycosaminoglycan biosynthesis - keratan sulfate	0.25326238
109	239	path:hsa00520	Amino sugar and nucleotide sugar metabolism	0.25197333

Continued

110	103	path:hsa04142	Lysosome	0.25170888
111	173	path:hsa00982	Drug metabolism - cytochrome P450	0.25116403
112	136	path:hsa04360	Axon guidance	0.25113678
113	260	path:hsa03050	Proteasome	0.25085394
114	35	path:hsa00240	Pyrimidine metabolism	0.25076955
115	30	path:hsa04350	TGF-beta signaling pathway	0.25021957
116	125	path:hsa03013	RNA transport	0.2501358
117	208	path:hsa03320	PPAR signaling pathway	0.24971136
118	22	path:hsa00511	Other glycan degradation	0.24905125
119	165	path:hsa04964	Proximal tubule bicarbonate reclamation	0.24880669
120	168	path:hsa04915	Estrogen signaling pathway	0.24717818
121	227	path:hsa00910	Nitrogen metabolism	0.24680035
122	149	path:hsa05214	Glioma	0.24613474
123	40	path:hsa00563	Glycosylphosphatidylinositol(GPI)-anchor biosynthesis	0.24609017
124	12	path:hsa05223	Non-small cell lung cancer	0.24484153
125	169	path:hsa04270	Vascular smooth muscle contraction	0.24480738
126	140	path:hsa00100	Steroid biosynthesis	0.24337171
127	161	path:hsa00072	Synthesis and degradation of ketone bodies	0.24282517
128	38	path:hsa05211	Renal cell carcinoma	0.2425746
129	148	path:hsa05410	Hypertrophic cardiomyopathy (HCM)	0.24256141
130	216	path:hsa05206	MicroRNAs in cancer	0.24216451
131	220	path:hsa05160	Hepatitis C	0.24130456
132	7	path:hsa00380	Tryptophan metabolism	0.24071829
133	137	path:hsa00510	N-Glycan biosynthesis	0.24061145
134	139	path:hsa04152	AMPK signaling pathway	0.24036383
135	240	path:hsa04210	Apoptosis	0.23960804
136	224	path:hsa00600	Sphingolipid metabolism	0.23927281
137	141	path:hsa00514	Other types of O-glycan biosynthesis	0.23873378
138	134	path:hsa00270	Cysteine and methionine metabolism	0.23825966
139	158	path:hsa00071	Fatty acid degradation	0.23653308
140	222	path:hsa05012	Parkinson,s disease	0.23563759
141	93	path:hsa00640	Propanoate metabolism	0.23500998
142	67	path:hsa05414	Dilated cardiomyopathy	0.23493643
143	11	path:hsa00604	Glycosphingolipid biosynthesis - ganglio series	0.23450791
144	130	path:hsa04623	Cytosolic DNA-sensing pathway	0.23378337
145	111	path:hsa04540	Gap junction	0.23377764
146	212	path:hsa03440	Homologous recombination	0.23197988
147	10	path:hsa04750	Inflammatory mediator regulation of TRP channels	0.23142563
148	164	path:hsa04622	RIG-I-like receptor signaling pathway	0.23141904
149	144	path:hsa00450	Selenocompound metabolism	0.23135617
150	68	path:hsa04066	HIF-1 signaling pathway	0.22858626
151	3	path:hsa00020	Citrate cycle (TCA cycle)	0.22764413
152	9	path:hsa03460	Fanconi anemia pathway	0.22598664
153	245	path:hsa03040	Spliceosome	0.22578057
154	21	path:hsa04151	PI3K-Akt signaling pathway	0.22538818
155	219	path:hsa05202	Transcriptional misregulation in cancer	0.22475653
156	218	path:hsa05203	Viral carcinogenesis	0.224702
157	163	path:hsa04621	NOD-like receptor signaling pathway	0.22416213
158	142	path:hsa03420	Nucleotide excision repair	0.22375545
159	78	path:hsa04730	Long-term depression	0.22365317
160	124	path:hsa03015	mRNA surveillance pathway	0.22346458
161	132	path:hsa05120	Epithelial cell signaling in Helicobacter pylori infection	0.223081
162	160	path:hsa04020	Calcium signaling pathway	0.2230018
163	214	path:hsa05204	Chemical carcinogenesis	0.22292808
164	44	path:hsa00564	Glycerophospholipid metabolism	0.22235655
165	188	path:hsa04015	Rap1 signaling pathway	0.22121232

Continued

166	31	path:hsa00330	Arginine and proline metabolism	0.22105706
167	187	path:hsa04975	Fat digestion and absorption	0.21958521
168	119	path:hsa03018	RNA degradation	0.21526966
169	221	path:hsa05161	Hepatitis B	0.21524865
170	13	path:hsa05222	Small cell lung cancer	0.21514203
171	215	path:hsa04917	Prolactin signaling pathway	0.21501488
172	171	path:hsa00980	Metabolism of xenobiotics by cytochrome P450	0.21455704
173	74	path:hsa04144	Endocytosis	0.21404272
174	82	path:hsa05221	Acute myeloid leukemia	0.21402805
175	178	path:hsa04960	Aldosterone-regulated sodium reabsorption	0.21263368
176	223	path:hsa00603	Glycosphingolipid biosynthesis - globo series	0.21250508
177	194	path:hsa00062	Fatty acid elongation	0.21194349
178	184	path:hsa00900	Terpenoid backbone biosynthesis	0.21125651
179	225	path:hsa00601	Glycosphingolipid biosynthesis - lacto and neolacto series	0.21078662
180	156	path:hsa05110	Vibrio cholerae infection	0.20903207
181	79	path:hsa04530	Tight junction	0.20550351
182	172	path:hsa00983	Drug metabolism - other enzymes	0.20440956
183	167	path:hsa04370	VEGF signaling pathway	0.20404324
184	251	path:hsa05169	Epstein-Barr virus infection	0.20323758
185	237	path:hsa00350	Tyrosine metabolism	0.19987236
186	181	path:hsa00590	Arachidonic acid metabolism	0.19781674
187	54	path:hsa04520	Adherens junction	0.19690753
188	213	path:hsa05205	Proteoglycans in cancer	0.19666104
189	110	path:hsa01040	Biosynthesis of unsaturated fatty acids	0.19610619
190	162	path:hsa04620	Toll-like receptor signaling pathway	0.19552807
191	61	path:hsa00650	Butanoate metabolism	0.19542822
192	204	path:hsa04070	Phosphatidylinositol signaling system	0.19395942
193	170	path:hsa00260	Glycine, serine and threonine metabolism	0.19320771
194	118	path:hsa05166	HTLV-I infection	0.19314874
195	244	path:hsa04060	Cytokine-cytokine receptor interaction	0.192685
196	127	path:hsa04810	Regulation of actin cytoskeleton	0.19265902
197	18	path:hsa03060	Protein export	0.19203336
198	123	path:hsa00770	Pantothenate and CoA biosynthesis	0.19092875
199	200	path:hsa05145	Toxoplasmosis	0.1906931
200	157	path:hsa05142	Chagas disease (American trypanosomiasis)	0.18974601
201	60	path:hsa05219	Bladder cancer	0.18964668
202	17	path:hsa04510	Focal adhesion	0.18883421
203	243	path:hsa00030	Pentose phosphate pathway	0.18636583
204	2	path:hsa04610	Complement and coagulation cascades	0.18613305
205	86	path:hsa03008	Ribosome biogenesis in eukaryotes	0.18376063
206	176	path:hsa00750	Vitamin B6 metabolism	0.18366907
207	166	path:hsa03430	Mismatch repair	0.18283119
208	70	path:hsa04630	Jak-STAT signaling pathway	0.18280503
209	85	path:hsa00480	Glutathione metabolism	0.18264308
210	131	path:hsa04115	p53 signaling pathway	0.18247053
211	91	path:hsa04977	Vitamin digestion and absorption	0.18240582
212	241	path:hsa04064	NF-kappa B signaling pathway	0.18031603
213	87	path:hsa00760	Nicotinate and nicotinamide metabolism	0.1788466
214	209	path:hsa05131	Shigellosis	0.17873745
215	34	path:hsa05132	Salmonella infection	0.17858831
216	230	path:hsa04380	Osteoclast differentiation	0.17851441
217	41	path:hsa00562	Inositol phosphate metabolism	0.17516931
218	183	path:hsa05152	Tuberculosis	0.17185469
219	274	path:hsa05168	Herpes simplex infection	0.17178692
220	32	path:hsa00130	Ubiquinone and other terpenoid-quinone biosynthesis	0.16978725
221	8	path:hsa05020	Prion diseases	0.16752624

Continued

222	276	path:hsa00532	Glycosaminoglycan biosynthesis - chondroitin sulfate / dermatan sulfate	0.1668719
223	226	path:hsa05162	Measles	0.16428324
224	72	path:hsa00360	Phenylalanine metabolism	0.16308837
225	152	path:hsa00740	Riboflavin metabolism	0.16195028
226	242	path:hsa04062	Chemokine signaling pathway	0.16177148
227	64	path:hsa04110	Cell cycle	0.1607954
228	180	path:hsa00592	alpha-Linolenic acid metabolism	0.15939683
229	234	path:hsa05164	Influenza A	0.15867545
230	205	path:hsa00232	Caffeine metabolism	0.15847641
231	27	path:hsa04662	B cell receptor signaling pathway	0.15645272
232	33	path:hsa05133	Pertussis	0.1551987
233	179	path:hsa03030	DNA replication	0.1548013
234	206	path:hsa05100	Bacterial invasion of epithelial cells	0.14962944
235	279	path:hsa04914	Progesterone-mediated oocyte maturation	0.14797757
236	39	path:hsa05134	Legionellosis	0.14797697
237	145	path:hsa02010	ABC transporters	0.14199179
238	247	path:hsa00780	Biotin metabolism	0.14096309
239	26	path:hsa04660	T cell receptor signaling pathway	0.13978458
240	65	path:hsa04114	Oocyte meiosis	0.13970636
241	250	path:hsa03450	Non-homologous end-joining	0.13873144
242	109	path:hsa00460	Cyanoamino acid metabolism	0.13824715
243	113	path:hsa05143	African trypanosomiasis	0.1370672
244	122	path:hsa05146	Amoebiasis	0.13398293
245	28	path:hsa04664	Fc epsilon RI signaling pathway	0.13166859
246	117	path:hsa04611	Platelet activation	0.12960384
247	273	path:hsa04670	Leukocyte transendothelial migration	0.12908715
248	228	path:hsa05323	Rheumatoid arthritis	0.12810859
249	186	path:hsa04974	Protein digestion and absorption	0.12491936
250	29	path:hsa04666	Fc gamma R-mediated phagocytosis	0.12469275
251	81	path:hsa05322	Systemic lupus erythematosus	0.12400582
252	196	path:hsa05140	Leishmaniasis	0.12388785
253	76	path:hsa00471	D-Glutamine and D-glutamate metabolism	0.12237875
254	16	path:hsa04512	ECM-receptor interaction	0.12210762
255	191	path:hsa00290	Valine, leucine and isoleucine biosynthesis	0.12144465
256	129	path:hsa00670	One carbon pool by folate	0.12126919
257	263	path:hsa00920	Sulfur metabolism	0.11944576
258	84	path:hsa05321	Inflammatory bowel disease (IBD)	0.11388533
259	98	path:hsa04145	Phagosome	0.1131427
260	211	path:hsa05130	Pathogenic Escherichia coli infection	0.10588805
261	199	path:hsa05144	Malaria	0.10394065
262	195	path:hsa04650	Natural killer cell mediated cytotoxicity	0.1025008
263	235	path:hsa04640	Hematopoietic cell lineage	0.10160391
264	271	path:hsa04672	Intestinal immune network for IgA production	0.10060175
265	159	path:hsa05150	Staphylococcus aureus infection	0.08712216
266	272	path:hsa00730	Thiamine metabolism	0.08193033
267	14	path:hsa04514	Cell adhesion molecules (CAMs)	0.0805607
268	116	path:hsa05310	Asthma	0.07940406
269	69	path:hsa05416	Viral myocarditis	0.07402959
270	4	path:hsa04612	Antigen processing and presentation	0.07208948
271	96	path:hsa04940	Type I diabetes mellitus	0.06952087
272	5	path:hsa05340	Primary immunodeficiency	0.06841601
273	83	path:hsa05320	Autoimmune thyroid disease	0.06470609
274	197	path:hsa00061	Fatty acid biosynthesis	0.06104635
275	56	path:hsa05330	Allograft rejection	0.0576579
276	57	path:hsa05332	Graft-versus-host disease	0.05119566

Continued

277	95	path:hsa00400	Phenylalanine, tyrosine and tryptophan biosynthesis	0.04524044
278	126	path:hsa03010	Ribosome	0.04481333
279	62	path:hsa00300	Lysine biosynthesis	NA
280	77	path:hsa00472	D-Arginine and D-ornithine metabolism	NA
